# Associations between grip strength, brain structure, and mental health in > 40,000 participants from the UK Biobank

**DOI:** 10.1186/s12916-022-02490-2

**Published:** 2022-09-09

**Authors:** Rongtao Jiang, Margaret L. Westwater, Stephanie Noble, Matthew Rosenblatt, Wei Dai, Shile Qi, Jing Sui, Vince D. Calhoun, Dustin Scheinost

**Affiliations:** 1 Department of Radiology and Biomedical Imaging, Yale School of Medicine, New Haven, CT 06510 USA; 2 Department of Biomedical Engineering, Yale University, New Haven, CT 06520 USA; 3 Department of Biostatistics, Yale University, New Haven, CT 06520 USA; 4 Tri-institutional Center for Translational Research in Neuroimaging and Data Science (TReNDS), Georgia State University, Georgia Institute of Technology, and Emory University, Atlanta, GA 30303 USA; 5 Interdepartmental Neuroscience Program, Yale University, New Haven, CT 06520 USA; 6 Department of Statistics & Data Science, Yale University, New Haven, CT 06520 USA; 7 Child Study Center, Yale School of Medicine, New Haven, CT 06510 USA

**Keywords:** Grip strength, Cognitive functioning, Mental health, Brain plasticity, Grey matter volume

## Abstract

**Background:**

Grip strength is a widely used and well-validated measure of overall health that is increasingly understood to index risk for psychiatric illness and neurodegeneration in older adults. However, existing work has not examined how grip strength relates to a comprehensive set of mental health outcomes, which can detect early signs of cognitive decline. Furthermore, whether brain structure mediates associations between grip strength and cognition remains unknown.

**Methods:**

Based on cross-sectional and longitudinal data from over 40,000 participants in the UK Biobank, this study investigated the behavioral and neural correlates of handgrip strength using a linear mixed effect model and mediation analysis.

**Results:**

In cross-sectional analysis, we found that greater grip strength was associated with better cognitive functioning, higher life satisfaction, greater subjective well-being, and reduced depression and anxiety symptoms while controlling for numerous demographic, anthropometric, and socioeconomic confounders. Further, grip strength of females showed stronger associations with most behavioral outcomes than males. In longitudinal analysis, baseline grip strength was related to cognitive performance at ~9 years follow-up, while the reverse effect was much weaker. Further, baseline neuroticism, health, and financial satisfaction were longitudinally associated with subsequent grip strength. The results revealed widespread associations between stronger grip strength and increased grey matter volume, especially in subcortical regions and temporal cortices. Moreover, grey matter volume of these regions also correlated with better mental health and considerably mediated their relationship with grip strength.

**Conclusions:**

Overall, using the largest population-scale neuroimaging dataset currently available, our findings provide the most well-powered characterization of interplay between grip strength, mental health, and brain structure, which may facilitate the discovery of possible interventions to mitigate cognitive decline during aging.

**Supplementary Information:**

The online version contains supplementary material available at 10.1186/s12916-022-02490-2.

## Background

Identifying modifiable risk factors and the neurobiological underpinnings that preserve cognitive functioning has become a public health priority in an ever-increasing aging society [1]. Among potential candidates, handgrip strength, which is often assessed isometrically using a hydraulic hand dynamometer, serves as an easily administered and validated measure of muscle strength and overall health in clinical settings [2, 3]. Accumulating evidence shows that lower grip strength, as a proxy for muscle strength, imposes serious limitations on dependency, disability, and quality of life in older age [4, 5]. Individuals with weak grip strength are at higher risk of adverse health consequences like mobility, frailty, falls, hospitalization, and all-cause mortality [6, 7].

Epidemiological research has associated weaker grip strength with reduced cognitive functioning and increased risk for psychiatric conditions and dementia. For example, population-scale investigations reveal consistent associations between maximal grip strength and performance on cognitive tasks of verbal reasoning, reaction time, and working memory in both the general population and individuals with schizophrenia, bipolar disorder, or depression [8, 9]. This aligns with experimental studies, which have related greater grip strength to motivated behaviors, like effort and vigor, that are perturbed in psychotic and mood disorders [10, 11]. A recent community-based study also found that older adults in the lowest quantile of muscular strength have a higher likelihood of suffering from depressive symptoms and suicidal ideation [12]. Moreover, meta-analytic findings have consistently implicated the predictive value of grip strength for health outcomes, highlighting its crucial role as a clinically useful indicator for monitoring cognitive impairment and progression of neurodegenerative diseases [13–15].

Despite such potential, significant gaps remain in our knowledge of the links between grip strength and mental health. First, most studies on this topic have been either based on a small set of circumscribed cognitive domains or relied on relatively insensitive clinical measures, such as the mini-mental state examination [14], which may not detect subtle cognitive changes that occur in early stages of aging [16]. By comprehensively examining associations between grip strength and a wide repertoire of mental health-related outcomes (including cognitive functioning, anxiety/depression symptoms, subjective well-being, and life satisfaction), rather than only focusing on cognition, one could determine the sensitivity of grip strength to specific health domains. This, in turn, would aid early identification and intervention efforts for neurodegenerative disorders. Second, since existing studies are largely cross-sectional, the temporal associations between handgrip strength and mental health remain poorly characterized. Although many studies assume that baseline handgrip strength predicts future cognitive decline [17], others demonstrate the reverse association [18]. Moreover, a recent study of 5995 Korean participants confirmed a bi-directional relationship between grip strength and cognitive functions, suggesting the existence of common pathways underlying these two constructs [19].

While previous work has begun to unravel the relationship between grip strength and cognitive function during aging, relatively little attention has been paid to the underlying mechanism. Examining how the brain mediates the relationship between grip strength and cognitive function would advance mechanistic understanding of age-related health outcomes during senescence. More importantly, it may facilitate the discovery of novel interventions to mitigate cognitive decline during aging. In light of the well-documented evidence that MRI-derived measures of brain grey matter volume (GMV) serves as indicators of underlying neuropathological alterations in neurodegenerative disorders, emerging evidence has begun to establish the relevance of GMV to potential protective factors, including physical fitness, and muscular strength [20–22]. Nevertheless, associations between global or regional GMV and grip strength remain inconsistent [23], which may be partially attributed to low statistical power due to small sample sizes. Crucially, a large-scale investigation with both sensitive measures of behavioral outcomes as well as brain imaging indices is needed to comprehensively (1) establish the behavioral relevance of grip strength, (2) disentangle the directionality, (3) unravel the neurobiological correlates, and (4) examine the mediation role of these brain biomarkers.

To fill these gaps, we examine the behavioral and neural signatures of grip strength in one of the largest population-scale neuroimaging cohorts, the UK Biobank [24–26]. Using data from over 40,000 participants, we start by establishing how grip strength relates to a total of 30 mental health-related behavioral phenotypes. Based on longitudinal data, we further determine the directionality of these associations. We then investigate how grip strength is related to global and regional GMV and quantify the extent to which its neurobiological correlates are correlated with mental health outcomes. Finally, we examine whether GMV mediates any associations between grip strength and mental health outcomes.

## Methods

### Cohort and participants

The UK Biobank project is a population-scale, prospective cohort study of > 500,000 participants recruited from across the UK [26]. Between 2006 and 2010, all participants received a baseline assessment that collected a variety of phenotypic and health-related information (baseline visit) [24]. Since 2014, a subsample of participants has been invited back to four assessment centers for brain imaging and an extensive set of behavioral assessments (imaging visit). The baseline visit did not collect any brain imaging data, and some behavioral measures (e.g., matrix pattern completion, trail making, symbol digit substitution) were not available. Moreover, because a primary aim of the current study is to examine the neurobiological basis of grip strength and the mediation role of GMV, we restricted the main analysis to data from the imaging visit although there were more subjects at the baseline visit.

**Fig. 1:**
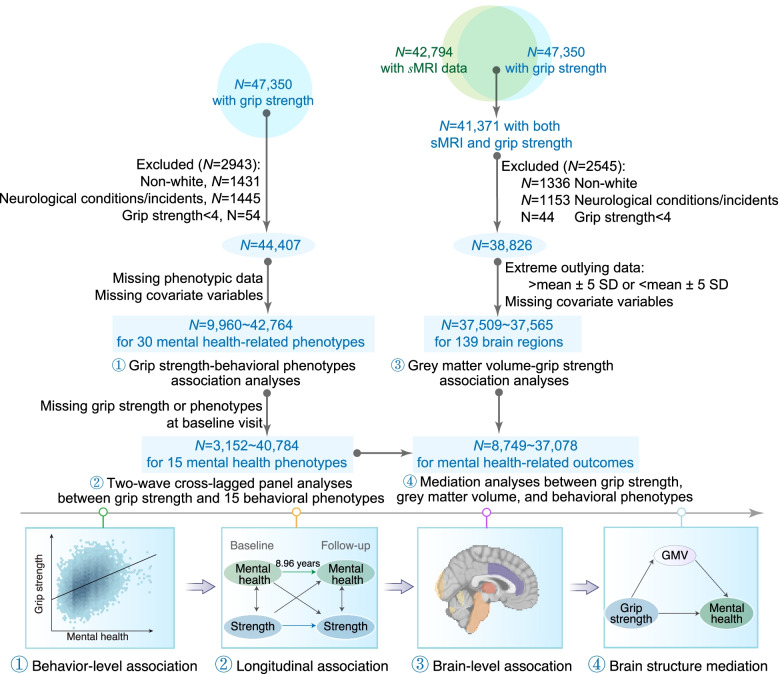
Flowchart illustrating criteria for selection of samples as well as the four analyses performed in the current study. Using data from over 40,000 participants, we start by establishing how grip strength relates to a total of 30 mental health-related behavioral phenotypes. Based on longitudinal data, we further determine the directionality of these associations. We then investigate how grip strength is related to regional GMV and examine whether GMV mediates any associations between grip strength and mental health outcomes

As per previous UK Biobank studies [8, 27, 28], we excluded participants who reported any of the listed severe neurological conditions/incidents (Additional file 1: Table S1). Additional exclusion criteria included missing MRI or relevant behavioral/demographic data. Since non-White participants constitute ~3% of this dataset, we only included White participants in the current study for further analysis. Overall, the behavioral analyses comprised a sample of 9960–42,764 participants per specific behavioral metric. In the longitudinal analyses, the number of participants was 3152–40,784 (age at baseline = 55.25±7.54 years, range = 40–70). In the brain imaging analyses, the number of participants was 37,509–37,565, 51% female, aged 64.21 ± 7.71 years (range: 45–82), and 48% had an education level of college (Fig. 1, and Additional file 1: Fig. S1).

### Data acquisition and preprocessing

T1-weighted MPRAGE data were acquired on a 3T Siemens Skyra scanner using a standard 32-channel head coil. The detailed parameters were: Resolution: 1 × 1 × 1 *mm*, field-of-view (FOV): 208 × 256 × 256 matrix, duration: 5 min. The estimates of GMV were processed and quality controlled by the UK Biobank team, and they were made available to approved researchers as image-derived phenotypes. Specifically, cortical tissue-type segmentation was completed using FAST (FMRIB’s Automated Segmentation Tool), and subcortical structures were segmented using FIRST (FMRIB’s Integrated Registration and Segmentation Tool) [29]. An extensive overview of the data acquisition protocols and preprocessing steps can be found at https://biobank.ctsu.ox.ac.uk/crystal/crystal/docs/brain_mri.pdf and elsewhere [25, 30]. Overall, a total of 139 brain regions were extracted (Additional file 1: Fig. S2). Extreme outlying data points (further than mean ± 5 SD) were excluded from the imaging analysis.

### Handgrip strength assessment

Following standard procedures [31], handgrip strength was assessed isometrically by a research assistant using a calibrated Jamar J00105 hydraulic hand dynamometer (Lafayette Instrument Company, IN, USA). With the participant seated upright with their elbow by their side and fixed at 90° (so that their forearm was facing forwards and resting on an armrest), a single trail indexing the maximal handgrip strength was acquired from each hand while allowing participants to select the most comfortable of 5 candidate grip positions [32]. In line with previous studies [8, 9], we used the reading from the self-reported dominant hand, or from the highest score from both hands if handiness is ambidextrous or unavailable. Participants with handgrip strength < 4 kg were identified as outliers and excluded from further analysis.

### Behavioral data assessment

The behavioral assessment battery was administered using a brief and fully automated touchscreen computer without supervision. Some of the cognition or mental health assessments were specifically developed for the UK Biobank while others were adapted from commonly used tests. We briefly describe these phenotypes and provide their field ID in the UK Biobank in the Additional file 1: Table S2 [33–36], and detailed information can be found at the UK Biobank website and elsewhere [37]. Overall, a total of 30 behavioral phenotypes were included in the current study based on their relevance to cognition and mental health. They can be categorized into 4 groups:

- Cognitive functioning (*n* = 17): fluid intelligence (reasoning), prospective memory, reaction time (processing speed), numeric memory (working memory), trail making (2 measures, executive function), symbol digit substitution (2 measures, processing speed), matrix pattern completion (non-verbal fluid reasoning), tower rearranging test (executive function), paired associate learning (verbal declarative memory), and pairs matching (6 measures, visual memory)
- Life satisfaction (*n* = 6): health satisfaction, family relationship satisfaction, friendship satisfaction, financial situation satisfaction, work/job satisfaction, and happiness
- Anxiety/depression (*n* = 4): neuroticism (12-item Eysenck Personality Questionnaire), depression symptoms (9-item Patient Health Questionnaire and the Composite International Diagnostic Interview [CIDI]), and anxiety symptoms (7-item Generalized Anxiety Disorder Questionnaire)
- Subjective well-being (*n*=3): “general happiness,” “happiness with own health,” and “belief life is meaningful”

### Association analysis between grip strength and 30 behavioral outcomes

Generalized linear mixed effect models (GLMMs) were employed to characterize how handgrip strength relates to each of the 30 behavioral outcomes adjusting for covariates, including age (in years), gender, education level, socioeconomic status (measured as Townsend deprivation index score), body mass index, height, and waist-to-hip ratio [32]. Following implementations in [8, 9], each behavioral phenotype was modeled as the response variable, and the grip strength and nuisance covariates were modeled as fixed effects. To account for the expected relatedness among data sites, the imaging site was modeled as a random effect, as recommended in studies using UK Biobank data [8, 9]. Depending on the distribution of behavioral phenotypes, LMM, GLMM with binomial error structure and logit link function, and GLMM with Poisson error structure were applied for continuous, binary (e.g., the prospective memory test) and count (e.g., the pairs-matching test: error made) phenotypes. Scores of behavioral phenotypes with significantly positive skew, like reaction time and trail making, were log-transformed. For the association analysis, we reported the relevant summary statistics, and associated two-tailed *P* values. Moreover, a Benjamini and Hochberg approach [38] was used to adjust for multiple comparisons to control false discovery rate (FDR), which was performed using the ‘p.adjust*’* function in R. Specifically, when testing for *m* hypotheses, this approach firstly orders all *P* values from lowest to highest, and then identifies the minimum index *k* such that P_FDR_=P_k_*m/k<significance level. Associations with FDR corrected *P*-values below 0.05 were considered as significant.

### Longitudinal association between grip strength and behavioral phenotypes

Among all 30 behavioral measures, 15 have baseline data that were collected approximately 8.96 ± 1.82 years prior to MRI scanning (*N* = 3152–40,784). As in prior studies [39, 40], a classic two-wave cross-lagged panel model [6] was estimated using structural equation modeling in *Mplus* (version 8.3) [41] to determine the longitudinal relationship between grip strength and behavioral outcomes. Specifically, the model examines the relative strength of the cross-lagged correlations between baseline grip strength and each of the 15 phenotypes at 10-year follow-up, as well as between baseline behavioral measures and subsequent grip strength, while adjusting for confounding covariates and the baseline behavioral or grip strength measurements. The model was estimated via maximum likelihood estimation with robust standard errors [39]. We report the standardized regression coefficients and standard errors.

### Association analysis of brain structure with grip strength and behavioral outcomes

We used the same analytical framework as described above to assess how regional GMV related to grip strength and behavioral outcomes while simultaneously accounting for confounders. To test whether associations between individual regional GMV and the grip strength or behavioral outcomes were confounded by the total intracranial volume (ICV), we repeated the GLMM analysis by additionally including the total ICV as a covariate. To investigate whether grip strength and 30 behavioral measures have common association maps, we calculated the Pearson’s correlation coefficient of the *t*-statistic maps between grip strength and each behavioral measure [42].

### Mediation analysis

In light of the strong associations between GMV and both grip strength and the behavioral outcomes (see the “Results” section), mediation analysis was performed to examine whether the association can be explained by differences in brain structure while adjusting for confounders [43]. We first calculated the mean GMV of brain regions that were significantly associated with grip strength. Then, mediation analysis was performed using the “mediation” package in R (version 4.1.2) [44]. Specifically, grip strength was used as an independent variable, and each of the behavioral phenotypes was used as a dependent variable. The mean GMV constituted the mediator. The mediation analysis was only performed on grip strength-associated behavioral phenotypes. For behavioral phenotypes whose values showed a longitudinal association with subsequent grip strength, the role of predictor and response variable was exchanged. The same confounding variables as used in the association analysis were controlled here. The significance of the mediation effects was assessed based on 5000 bootstrap iterations.

## Results

### Behavioral relevance of grip strength to mental health

**Fig. 2:**
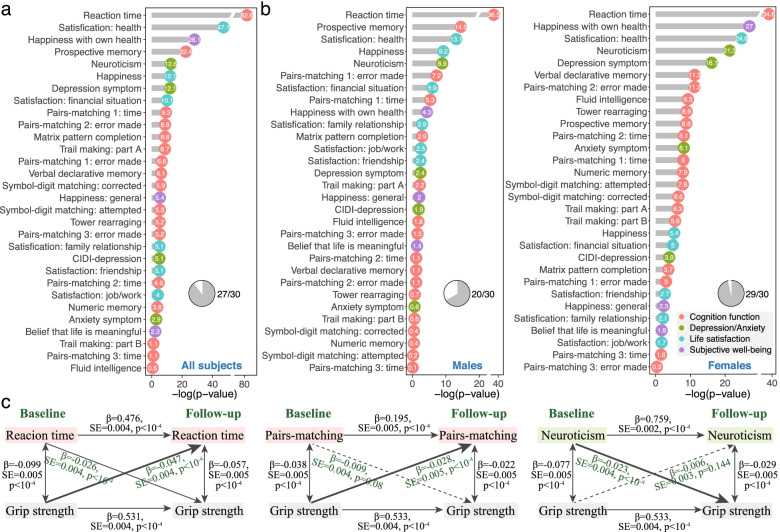
Associations between handgrip strength and 30 mental health-related outcomes. **a** Of the 30 behavioral phenotypes, 27 showed significant association with grip strength and in the expected direction after controlling for confounders: stronger muscular strength was associated with better cognitive performance, higher life satisfaction, greater subjective well-being, and lower depression and anxiety symptoms. Significance is shown as -log_10_(FDR corrected *P*-value) and a value above 1.30 is considered statistically significant (-log_10_(0.05) = 1.30). **b** When the analyses were stratified by gender, a respective of 29 and 20 behavioral outcomes showed significant association with grip strength in females and males. **c** Three examples of the longitudinal association between grip strength and behavioral outcomes were revealed by a classic two-wave cross-lagged panel model. For the reaction time test, we observed a significant bi-directional association, i.e., stronger grip strength at baseline was related to better performance on reaction time at the 9-year follow-up, while the reverse was weaker but also significant (FDR corrected *P* < 10^−4^); for pairs matching, greater grip strength predicts higher task performance, while the reverse was nonsignificant; For neuroticism, a higher neuroticism score was associated with weaker grip strength measured 9 years later, but the reverse was nonsignificant

The behavioral analysis included a maximum of 42,843 participants who completed at least one mental health assessment. In general, increased grip strength were associated with young age (*r* = − 0.156, *P* < 10^−10^), male gender (*t* = 205.74, *P* < 10^−10^), above college education (*t* = 11.99, *P* < 10^−10^), low socioeconomical score (*r*=-0.019, *P* = 6.67 × 10^−5^), and with increasing height (*r*=0.658, *P* < 10^−10^), body mass index (*r*=0.097, *P* < 10^−10^), and waist-to-hip ratio (*r* = 0.418, *P* < 10^−10^). Among all covariates, gender showed the greatest association with grip strength. Figure 2a and Additional file 1: Table S3 display the results of the GLMMs in examining the association of grip strength with each of the 30 behavioral phenotypes. Of all these outcomes, 27 were significantly correlated with grip strength while controlling for confounding variables (FDR corrected *P* < 0.05). All correlations were in the expected direction, with stronger grip strength relating to improved cognitive performance, higher life satisfaction, greater subjective well-being, and lower depression and anxiety symptoms. The strongest effect for the behavioral outcomes was observed for reaction time (*N* = 40,278, *t* = − 19.56, FDR corrected *P* = 2.42 × 10^−83^) and followed by health satisfaction (*N* = 42,764, *t* = − 14.74, *P* = 7.16 × 10^−48^), happiness with own health (*N* = 29,501, *t* = − 11.35, *P* = 8.11 × 10^−29^), and prospective memory (*N* = 40,530, *t* = 10.12, odds ratio=1.27, *P* = 3.75 × 10^−23^, Additional file 1: Fig. S3). Negative effects were due to behavior task scoring where higher scores index worse performance.

When the behavioral analyses were stratified by gender, similar results were found, with the association patterns highly correlated between males and females (*r* = 0.81, *P* = 6.64 × 10^−8^, Fig. 2b). However, the associations in females were generally stronger than those in males, and more behavioral outcomes showed significant associations with grip strength in females (*N* = 29) than in males (*N* = 20).

### Longitudinal association between grip strength and mental health

Additional file 1: Fig. S4 details full results of the classic two-wave cross-lagged panel model in examining the longitudinal associations between grip strength and 15 mental health outcomes. Overall, results showed that poorer grip strength at baseline was a significant predictor of decreased cognitive performance in fluid intelligence (*β* = 0.019, FDR corrected *P* = 0.041), prospective memory (*β* = 0.025, *P* = 0.007), reaction time (*β* = − 0.047, *P* < 10^−4^), and pairs matching (*β* = − 0.028–*β* =− − 0.016, *P* < 0.003) at the 9-year follow-up after controlling for confounders and the corresponding baseline cognitive measure, but not for numeric memory (*P* = 0.952). The reverse was weaker and a significant bi-directional relationship was only found for reaction time (*β* = − 0.026, *P* < 10^−4^). Conversely, the analysis revealed a significant path from baseline mental status measures of neuroticism (*β* = − 0.023, *P* < 10^−4^), health satisfaction (*β* = − 0.025, *P* < 10^−4^), and financial situation satisfaction (*β* = − 0.024, *P* = 0.003) to subsequent grip strength, while the reverse was nonsignificant (Fig. 2c).

### Neural signatures of grip strength and mental health outcomes

**Fig. 3:**
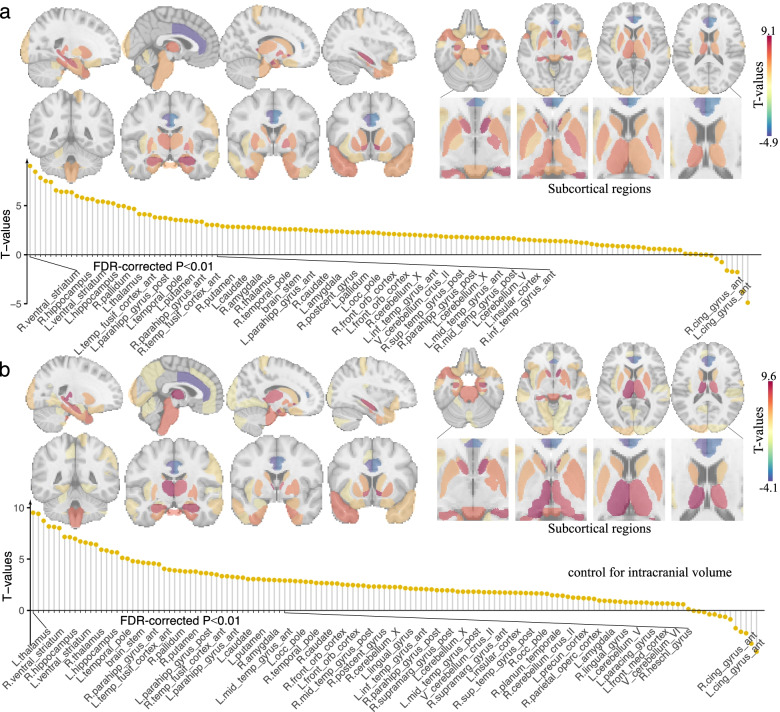
Regional distribution of associations between grey matter volume and grip strength. **a** The regional analysis revealed widespread significant associations between grip strength and grey matter volume after controlling for potential confounders. T-statistics are visualized here. **b** The associations remained significant (FDR corrected *P* < 0.01) after additionally controlling for total intracranial volume, implying that grip strength relates to region GMV independently of overall brain volume. Brain regions showing the highest correlations with grip strength primarily included the ventral striatum, hippocampus, thalamus, temporal pole, parahippocampal gyrus, temporal fusiform cortex, brain stem, pallidum, and putamen

The regional analysis also revealed widespread significant associations between stronger grip strength and increased GMV (FDR corrected *P* < 0.01, Fig. 3a, Additional file 1: Table S4). When covarying for total ICV and the squared ICV, the results remained nearly unchanged (Fig. 3b, Additional file 1: Table S5), with the association maps highly correlated between the cases with and without adding the total ICV and ICV^2^ as covariates (*r* = 0.96, *P* < 10^−30^, Additional file 1: Fig. S5), suggesting that associations between grip strength and GMV reflect local variation in GMV across the cortex as opposed to overall brain size. Brain regions showing the highest correlations with grip strength primarily included: the ventral striatum, hippocampus, thalamus, temporal pole, parahippocampal gyrus, temporal fusiform cortex, brain stem, pallidum, and putamen. Regional distribution of associations between GMV and grip strength for males and females can be found in Additional file 1: Fig. S6 and S7.

**Fig. 4:**
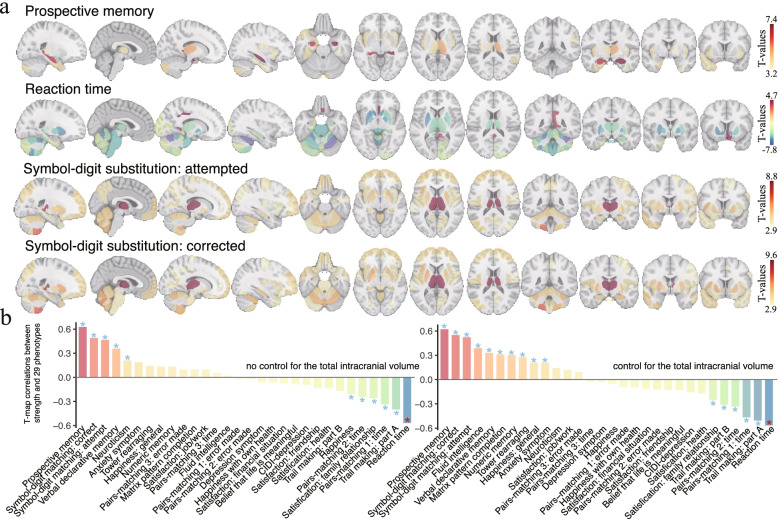
Regional distribution of associations between grey matter volume and cognitive function. **a** Significant associations were observed between behavioral phenotypes and regional grey matter volume after controlling for potential confounders (FDR corrected *P* < 0.01). The top 4 behavioral phenotypes showing the highest similarities of association map with grip strength were visualized here. **b** Comparison between GMV-grip strength and GMV-behavior association maps. The brain association map of grip strength was highly similar to that of the behavioral phenotypes regardless of whether the total intracranial volume was added as a covariate. The T-statistic map correlations reach statistical significance in 11 and 16 of all 30 behavioral outcomes in cases of including or not including the total intracranial volume as an additional covariate (*FDR corrected *P* < 0.05)

The associations between region-wise GMV and each of the 30 behavioral outcomes were also in the expected direction, regardless of whether the total intracranial volume was added as a covariate (Fig. 4a, Additional file 1: Fig. S8). Moreover, the association map of grip strength was significantly similar to that of the behavioral phenotypes, with absolute correlations ranging from 0.024 to 0.620 (16 of which reach statistical significance, FDR corrected *P* < 0.05, Fig. 4b). The top 4 behavioral phenotypes showing the highest similarities of association map with grip strength were prospective memory, reaction time, symbol-digit substitution: corrected, and symbol-digit substitution: attempted (Additional file 1: Fig. S9).

### Mediation effect of GMV

**Fig. 5:**
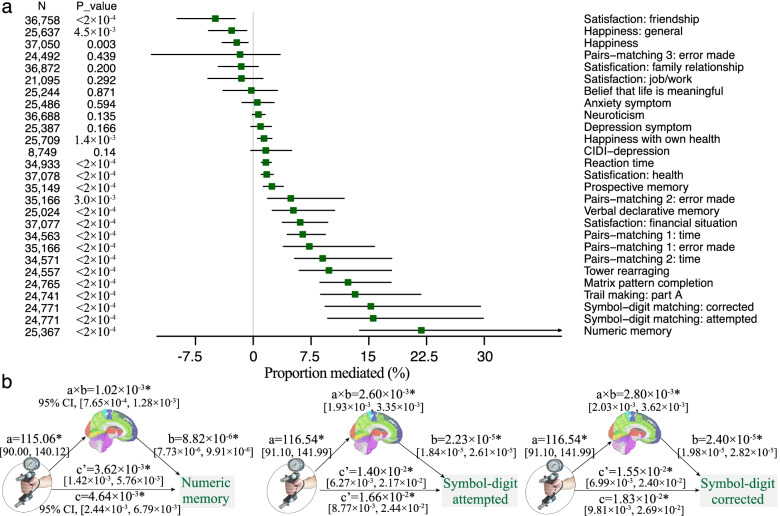
Mediation effects of brain volume on the association between grip strength and behavioral outcomes. **a** Mediation effect of mean GMV on the association between grip strength and behavioral outcomes. The proportion of variance explained by the mediation as well as the lower and upper bound of 95% confidence interval was shown. **b** The top three behavioral phenotypes having the highest mediation effect size were numeric memory (proportion mediated = 21.83%; 95% CI = 13.80%~41.88%; bootstrapping test, FDR corrected *P* < 2 × 10^−4^), symbol-digit substitution: attempted (15.58%; 9.66%~29.87%; *P* < 2 × 10^−4^), and symbol-digit substitution: corrected (15.26%; 9.31%~29.51%; *P* < 2 × 10^−4^). a×b: the indirect effect; c: the total effect; c’: the direct effect

The mean GMV of the significant brain regions shown in Fig. 3 was highly correlated with grip strength (*r* = 0.485, *P* < 10^−30^). Mediation analyses revealed a significant indirect effect (a×b) of the mean GMV in 16 of all 27 behavioral outcomes (FDR corrected *P* < 0.05, the proportion of mediated effect size ranged from 1.40% to 21.83%, Fig. 5a, Additional file 1: Table S6), indicating that the brain GMV significantly and partially mediated the relationship between grip strength and some of the mental health outcomes. Specifically, the top three behavioral measures having the highest mediation effect size were numeric memory (proportion mediated = 21.83%), symbol-digit substitution: attempted (15.58%), and symbol-digit substitution: corrected (15.26%, Fig. 5b). Mediation effects based on the first principal component of 139 brain regions were provided in Additional file 1: Fig. S10.

For females, of 29 behavioral outcomes that were significantly associated with grip strength, mediation analyses revealed a significant indirect effect of the mean GMV in 18 behaviors (FDR corrected *P* < 0.05, Additional file 1: Fig. S6). For males, the mean GMV significantly mediated the association between grip strength and 8 of 20 behavioral outcomes (FDR corrected *P* < 0.05, Additional file 1: Fig. S7).

## Discussion

Using one of the largest available datasets (> 40,000 participants), this study dissected the complex interplay between grip strength, behavioral outcomes, and brain structure. We replicated and extended established relationships between stronger handgrip strength and better mental health, both cross-sectionally and longitudinally. Furthermore, we identified novel associations between grip strength and greater GMV across basal ganglia and limbic regions, and we characterized how patterns of regional GMV associated with grip strength were related to various mental health outcomes. Moreover, we demonstrated that mean regional GMV considerably mediates the association between grip strength and several measures of cognition and mental health.

The reported link between grip strength and cognition aligns with previous studies [13, 15, 45], which suggests that grip strength may serve as a complementary measure of cognitive ability in aging adults. Going beyond limited domains or insensitive cognitive measures, we investigated a broader constellation of cognitive metrics spanning domains of memory, executive function, reasoning, and processing speed that are sensitive to subtle, early changes related to aging. In addition, we investigated several behavioral measures that are closely related to health status but have never been directly related to grip strength [12]. Of the 30 behavioral outcomes examined here, reaction time, which reflects cognitive domains of processing speed [37], demonstrated the most robust association with grip strength. As suggested by Joesh et al. [8], the strong association may be partially explained by the high dependence of the reaction time task on motor speed and dexterity, which are closely related to muscular function of hands. Further, in contrast to other behavioral outcomes that were based on self-report, the cognitive task-measured reaction time scores may be more informative in capturing inter-individual variability. Moreover, according to the general slowing theory, the decline in reaction time is a leading and sensitive indicator of cognitive aging and can lead to decrements in other domains like executive functions and working memory [46]. Deficits in processing speed have been observed across many psychiatric disorders, including schizophrenia and other neurodevelopmental disorders [47, 48], and recent work in the UK Biobank suggests that the association between grip strength and reaction time is weaker in individuals with schizophrenia, depression, and bipolar disorder relative to the general population [8, 9]. In addition, we found that the associations between grip strength and mental health outcomes were stronger in females than in males, implying distinct mechanisms between them. Consistently, a recent study [49] based on Mendelian randomization analysis also identified shared pathways between grip strength and depression in females but not in males. In light of this, it is of interest for future investigations to ascertain any gender specificity of the beneficial effects stemming from physical exercise aiming at enhancing muscular fitness.

These findings increased current understanding on the use of grip strength not only as a proxy of physical fitness but also as a malleable indicator for health status in detecting early impairment of specific domains. Unsurprisingly, some of the associations were small and likely due in part to the fact that we controlled for numerous demographic, socioeconomic, and anthropometric measures that are expected to co-occur with health status [50, 51] and muscular strength [27]. Consequently, the association effects should be interpreted as the relevance of grip strength to these mental health outcomes beyond the contribution of covariates [28]. Moreover, this result also implies the power of large samples in identifying subtle effects that may not be detectable in smaller samples.

Our study also provides evidence for the directionality of the connection between grip strength and mental health. Specifically, the longitudinal analyses showed that baseline grip strength was related to cognitive performance at ~9 years follow-up, while the reverse effect was much weaker, and a significant bi-directional relationship was only found for reaction time. This is consistent with most longitudinal findings that implicate stronger grip strength as a protective factor against cognitive decline and dementia [13, 14, 52, 53] but also supports the notion that changes in these two constructs parallel each other over time [19, 54]. This finding supports the utility of grip strength as a potential treatment target for improved cognitive outcomes in older adults. Indeed, a recent intervention study found that 6 months of resistance training, but not computerized cognitive training, can significantly improve global cognition in older patients with dementia [55]. The bi-directional relationship was also encouraging as they implied that interventions that enhance either muscular fitness or cognitive capabilities may generate beneficial effects on the other [3, 19]. Furthermore, we demonstrated significant paths from baseline neuroticism, health and financial satisfaction to subsequent grip strength. As these measures were pertinent to mental health and life satisfaction, it may suggest that people with greater resilience and satisfaction were more likely to engage in physical activities [19]. Together, the behavioral analyses emphasized the need for increased awareness in clinical practice to incorporate muscular strength into routine assessment and provided insights into possible interventions to prevent cognitive decline during aging.

At the brain level, grip strength revealed mostly significant positive correlations with brain volume, which aligns with previous evidence at the global level [23]. As the global GMV is a general reflection of health status and GM atrophy is a signature of neurodegeneration [27, 28], the identified association pattern may suggest that having stronger muscular strength also relates to better overall brain health [22]. Furthermore, given that many regional GM associations reach a significant effect after controlling for the total ICV, it implies that grip strength may exert a region-specific beneficial effect on brain structure. Specifically, prominent among these select regions were the ventral striatum, hippocampus, thalamus, temporal pole, parahippocampal gyrus, pallidum, and putamen, evidencing the possibility that GMV underlies individual differences in muscular strength. These findings accord with previous evidence suggesting the crucial role these subcortical, limbic (especially the hippocampus), and temporal cortices play in muscular fitness [23, 56, 57].

In addition, we showed that the brain association map of grip strength was highly similar to that of the behavioral phenotypes and that the GMV significantly and partially mediated their associations. These findings raised the possibility that common neurobiological pathways underlie individual differences in grip strength and these behavioral outcomes [58]. Indeed, this hypothesis has gained support from neuroimaging studies linking higher grip strength and better cognitive performance to greater brain volume. For instance, a cross-sectional study in 835 older adults observed that weakness in strength was associated with reduced GMV in the hippocampus and fusiform cortex, which were implicated in high-order cognitive processing and social functioning [57]. Similarly, subcortical nuclei degeneration may underlie the pathogenesis of “cognitive frailty,” which was defined as the simultaneous presence of cognitive decline and physical frailty [56]. Also supportive is the finding showing the enhancement of brain plasticity in older adults following physical training [55, 59–61]. Specifically, Suo et al. showed that 6-month resistance training improved not only overall cognitive performance but also elicited GM expansion for participants at risk for dementia [55]. This study, together with ours, suggests that the relationship between muscular fitness and mental health may be mediated by increased GMV in regions having high plasticity like the hippocampus.

The present findings are consistent with existing evidence of how musculoskeletal strength relates to brain health. Skeletal muscle plays a crucial role in the production and secretion of many cytokines such as brain-derived neurotrophic factor (BDNF) and insulin-like growth factor-1 [14], which are involved in neuronal survival, synaptic development, angiogenesis, learning, and neural plasticity [62]. Higher levels of these peptides have been correlated with greater physical fitness and increased GMV [58], and more importantly, studies showed that resistance activities could stimulate the release of BDNF and evoke neuroplastic changes in frontal and hippocampal regions, which may further translate into cognitive improvements [63–65]. Therefore, cytokine-induced alterations in GMV may represent a mechanism through which muscular fitness influences cognition and mental status, yet further research is needed [66].

The results from both cross-sectional and longitudinal analyses indicated a significant association between grip strength and cognitive functioning and mental status. This implies that grip strength can be used as a complementary measure of mental health in aging adults and the routine assessment should be recommended in clinical practice. The large sample size (*N* > 40,000), sufficient control of confounders (including demographic, anthropometric, and socioeconomic covariates), use of multiple-comparisons correction, subgroup sensitivity analyses, and the longitudinal design ensure our current results are reliable and less likely to suffer from replication failure [67]. Moreover, our results regarding the association between grip strength, mental health, and brain structure are mostly consistent with existing small-sampled studies. There are some limitations to be acknowledged. First, we report statistical mediation effects that are strictly measures of association [39, 68], and causal inferences cannot be drawn from these models without further validation using randomized controlled trials. Nevertheless, these analyses represent a critical first step in characterizing associations between grip strength, brain structure, and mental health that can be further explored in longitudinal studies. To facilitate the use of grip strength in clinical settings, examining how interventions to enhance muscular strength would influence cognition capacities and brain health, especially in people with psychiatric disorders, is necessary. Second, as even small effects can reach statistical significance in a large sample, the magnitude of association may not be directly translated into clinical utility [69]. Third, as noted by Genon et al. [70], brain structure-behavior association studies are suffering from a replication crisis, where a poor replicability has been shown in both behavioral measurements and brain structure estimates. As such, reliability and replicability of the current findings merits further examination in external cohorts with great diversity in geographic, demographic, and sociocultural aspects. Further, going beyond statistical univariate approaches, further studies can take into account the multivariate nature of structural and behavioral measurements by leveraging machine learning techniques within cross-validated frameworks [71–73]. For in-depth discussion of this topic, we point the interested reader to [70]. Forth, some of the behavioral outcomes were assessed by ordinal measures, which represent different levels of fidelity [28]. It is possible that assessing these ordinal outcomes using continuous measures would prove more informative. Finally, there may be some other behavioral phenotypes that are related to health outcomes in the UK Biobank but not examined here. Future studies can examine the associations of grip strength with these less-commonly used outcomes.

## Conclusions

In sum, the current study showed that stronger grip strength was associated with better mental health, cross-sectionally and longitudinally. At the brain level, we found widespread associations between grip strength and greater GMV in subcortical and temporal cortices. Moreover, these GMV also correlated with better mental health and considerably mediated the effect of grip strength on cognitive functioning. Overall, our finding provides insights into the complex interplay between grip strength, mental health, and brain structure.

## Supplementary Information

**Additional file 1: Table S1.** Neurological conditions/incidents that are used to exclude participants in UK Biobank. **Table S2.** UK Biobank cognition and mental health measures used in the current study. **Table S3.** Association between grip strength and behavioral phenotypes. **Table S4.** Brain regions showing significant correlations with grip strength. **Table S5.** Brain regions showing significant correlations with grip strength after controlling for the total intracranial volume. **Table S6.** Results of the mediation analyses between grip strength, phenotypes, and mean grey matter volume. **Fig. S1.** A brief summary of the population characteristics of all participants used in the current study. **Fig. S2.** The 139 brain regions and their names. **Fig. S3.** The top four behavioral outcomes showing the strongest associations with grip strength. **Fig. S4.** Results of the classic two-wave cross-lagged panel model for 15 behavioral phenotypes that have complete data at two time points. **Fig. S5.** The correlation of association maps (T-maps) between the cases with and without including the total intracranial volume (ICV) and the squared ICV as covariates in examining the association of grip strength with regional grey matter volume across 139 regions. **Fig. S6.** Regional distribution of associations between grey matter volume and grip strength and the mediation effect of mean GMV in females. **Fig. S7.** Regional distribution of associations between grey matter volume and grip strength and the mediation effect of mean GMV in males. **Fig. S8.** The correlation of T-maps between the cases with and without including the total intracranial volume as a covariate in examining the association of grey matter volumes with behavioral outcomes across 139 regions. **Fig. S9.** Regional distribution of associations between grey matter volume and four representative behavioral phenotypes. **Fig. S10.** Mediation effects of the first principal component of 139 regional GMV on the association between grip strength and behavioral outcomes.

## Data Availability

All data used in this study are publicly accessible from UK Biobank via their standard data access procedure at https://www.ukbiobank.ac.uk/. Researchers can apply for access to the UK Biobank data via the Access management System (AMS) (https://www.ukbiobank.ac.uk/enable-your-research/apply-for-access). Code used in the current study is available from the authors upon reasonable request and can be found at can be found at https://github.com/Jiang-brain/Grip-strength-association.

## Abbreviations

BDNF: Brain-derived neurotrophic factor
FDR: False discovery rate
GLMM: Generalized linear mixed effect model
GMV: Grey matter volume
ICV: Intracranial volume
LMM: Linear mixed effect model
