# Supplementary material for: Associations between grip strength, brain structure, and mental health in > 40,000 participants from the UK Biobank
Source: BMC Med. 2022 Sep 9;20:286. doi: 10.1186/s12916-022-02490-2 (PMC9461129; doi:10.1186/s12916-022-02490-2)
Supplement: Supplementary file 1 — Additional file 1: Table S1. Neurological conditions/incidents that are used to exclude participants in UK Biobank. Table S2. UK Biobank cognition and mental health measures used in the current study. Table S3. Association between grip strength and behavioral phenotypes. Table S4. Brain regions showing significant correlations with grip strength. Table S5. Brain regions showing significant correlations with grip strength after controlling for the total intracranial volume. Table S6. Results of the mediation analyses between grip strength, phenotypes, and mean grey matter volume. Fig. S1. A brief summary of the population characteristics of all participants used in the current study. Fig. S2. The 139 brain regions and their names. Fig. S3. The top four behavioral outcomes showing the strongest associations with grip strength. Fig. S4. Results of the classic two-wave cross-lagged panel model for 15 behavioral phenotypes that have complete data at two time points. Fig. S5. The correlation of association maps (T-maps) between the cases with and without including the total intracranial volume (ICV) and the squared ICV as covariates in examining the association of grip strength with regional grey matter volume across 139 regions. Fig. S6. Regional distribution of associations between grey matter volume and grip strength and the mediation effect of mean GMV in females. Fig. S7. Regional distribution of associations between grey matter volume and grip strength and the mediation effect of mean GMV in males. Fig. S8. The correlation of T-maps between the cases with and without including the total intracranial volume as a covariate in examining the association of grey matter volumes with behavioral outcomes across 139 regions. Fig. S9. Regional distribution of associations between grey matter volume and four representative behavioral phenotypes. Fig. S10. Mediation effects of the first principal component of 139 regional GMV on the association between grip strength and behavio [file 12916_2022_2490_MOESM1_ESM.docx]

**Supplemental Material**

**Associations between grip strength, brain structure, and mental health in >40,000 participants from the UK Biobank**

| **Table S1. Neurological conditions/incidents that are used to exclude participants in UK Biobank** |
| --- |
| - Acute infective polyneuritis/guillain-barre syndrome - Benign neuroma - Brain abscess/intracranial abscess - Brain haemorrhage - Cerebral aneurysm - Cerebral palsy - Chronic/degenerative neurological problem - Dementia/Alzheimer's disease/cognitive impairment - Encephalitis - Epilepsy - Head injury - Infection of nervous system - Ischaemic stroke - Meningioma/benign meningeal tumor - Meningitis - Motor neurone disease - Multiple sclerosis - Neurological injury/trauma - Other demyelinating condition - Other neurological problem - Parkinson's disease - Spina bifida - Stroke - Subarachnoid haemorrhage - Subdural haemorrhage/haematoma - Transient ischaemic attack (tia) |

| **Table S2. UK Biobank cognition and mental health measures used in the current study** |
| --- |
| **Cognition functioning:**   - Fluid intelligence [*Field ID*: 20016]: verbal and numerical reasoning multiple-choice questions answered correctly in two minutes (total=13). - Prospective memory [*Field ID*: 20018]: dichotomous variable assessing whether participants succeeded to perform a planned action after a delayed period. - Reaction time [*Field ID*: 20023]: mean time (across four trials), in milliseconds, to press the button-box when two presented cards matched in a Go/No-Go test - Numeric memory [*Field ID*: 4282]: the maximum number of digits correctly remembered in the reverse order before two failures or up to 12 digits. - Trail making [*Field ID*: 6348, 6350]: the time in deci-seconds to complete Path A (numeric path) and Path B (alphanumeric path). - Symbol digit substitution [*Field ID*: 23323, 23324]: the number of attempted and correct symbol-digit matches made in 60 seconds. - Matrix pattern completion [*Field ID*: 6373]: the number of puzzles (non-verbal fluid reasoning) correctly solved in 3 minutes (total=15). - Tower rearranging test [*Field ID*: 21004]: the number of items answered correctly in 3 minutes, where for each item the participant indicates how many moves are required to match two displays. - Paired associate learning [*Field ID*: 20197]: the number of word pairs correctly associated (total=10). - Pairs matching [*Field ID*: 399-1, 399-2, 399-3, 400-1, 400-2, 400-3]: the number of attempts as well as the completion time required to correctly match 3, 6, 8 pairs of symbol cards following a brief visual presentation of these stimuli.   **Neuroticism, depression/anxiety:**   - Neuroticism [*Field ID*: 1920, 1930, 1940, 1950, 1960, 1970, 1980, 1990, 2000, 2010, 2020, 2030]: the proportion of ‘yes’ responses completed in a 12-item Eysenck Personality Questionnaire (Participants completing <9 items were excluded from further analysis). - Depression [*Field ID*: 20507, 20508, 20510, 20511, 20513, 20514, 20517, 20518, 20519]: the sum score from the 9-item Patient Health Questionnaire, which was taken during the online follow-up. - Anxiety [*Field ID*: 20505, 20506, 20509, 20512, 20515, 20516, 20520]: the sum score from the 7-item Generalized Anxiety Disorder Questionnaire, which was taken during the online follow-up. - CIDI depression [*Field ID*: 20441, 20446, 20449, 20532, 20536, 20435, 20437, 20450]: the depression section of the Composite International Diagnostic Interview Short Form (CIDI-SF).   **Subjective well-being:**   - General happiness [*Field ID*: 20458]: response to the question: "In general how happy are you?", which was taken during the online follow-up and coded as 1 (extremely happy) to 6 (extremely unhappy). - Happiness with own health [*Field ID*: 20459]: response to the question: "In general how happy are you with your HEALTH?", which was taken during the online follow-up and coded as 1 (extremely happy) to 6 (extremely unhappy). - Belief life is meaningful [*Field ID*: 20460]: response to the question: "To what extent do you feel your life to be meaningful?", which was taken during the online follow-up and coded as 1 (Not at all) to 5 (An extreme amount).   **Life satisfaction:**   - Health satisfaction [*Field ID*: 4548]: response to the question: "In general how satisfied are you with your HEALTH?", which was coded as 1 (extremely happy) to 6 (extremely unhappy). - Family relationship satisfaction [*Field ID*: 4559]: response to the question: "In general how satisfied are you with your FAMILY RELATIONSHIPS?", which was coded as 1 (extremely happy) to 6 (extremely unhappy). - Friendship satisfaction [*Field ID*: 4570]: response to the question: "In general how satisfied are you with your FRIENDSHIPS?", which was coded as 1 (extremely happy) to 6 (extremely unhappy). - Financial situation satisfaction [*Field ID*: 4581]: response to the question: "In general how satisfied are you with your FINANCIAL SITUATION?", which was coded as 1 (extremely happy) to 6 (extremely unhappy). - Work/job satisfaction [*Field ID*: 4537]: response to the question: "In general how satisfied are you with the WORK that you do?", which was coded as 1 (extremely happy) to 6 (extremely unhappy). - Happiness [*Field ID*: 4526]: response to the question: "In general how happy are you?", which was coded as 1 (extremely happy) to 6 (extremely unhappy). |

| **Table S3. Association between grip strength and behavioral phenotypes** | | | | | |
| --- | --- | --- | --- | --- | --- |
|  | Total No. | Coeff | SE | t value | P_FDR_ |
| **Cognition function** | | | | | |
| Fluid intelligence | 39,673 | 0.008 | 0.007 | 1.127 | 2.60×10^-01^ |
| Prospective memory | 40,530 | 0.237 | 0.024 | 10.116 | 3.75×10^-23^ |
| Reaction time | 40,278 | -0.160 | 0.008 | -19.56 | 2.42×10^-83^ |
| Numeric memory | 29,510 | 0.032 | 0.009 | 3.733 | 2.28×10^-04^ |
| Trail making: part A | 28,745 | -0.056 | 0.009 | -6.148 | 1.99×10^-09^ |
| Trail making: part B | 28,031 | -0.015 | 0.009 | -1.747 | 8.65×10^-02^ |
| Symbol-digit substitution: attempted | 28,783 | 0.038 | 0.008 | 4.703 | 4.54×10^-06^ |
| Symbol-digit substitution: corrected | 28,783 | 0.041 | 0.008 | 4.964 | 1.39×10^-06^ |
| Matrix pattern completion | 28,774 | 0.064 | 0.010 | 6.198 | 1.63×10^-09^ |
| Tower rearranging | 28,538 | 0.041 | 0.009 | 4.635 | 5.98×10^-06^ |
| Verbal declarative memory | 29,085 | 0.048 | 0.009 | 5.097 | 7.44×10^-07^ |
| Pairs-matching 1: error made | 40,551 | -0.098 | 0.019 | -5.299 | 2.71×10^-07^ |
| Pairs-matching 2: error made | 40,551 | -0.03 | 0.005 | -6.192 | 1.64×10^-09^ |
| Pairs-matching 3: error made | 28,066 | -0.028 | 0.006 | -4.619 | 6.10×10^-06^ |
| Pairs-matching 1: time | 39,870 | -0.068 | 0.010 | -6.415 | 4.75×10^-10^ |
| Pairs-matching 2: time | 39,872 | -0.044 | 0.010 | -4.250 | 2.79×10^-05^ |
| Pairs-matching 3: time | 15,903 | -0.022 | 0.013 | -1.730 | 8.65×10^-02^ |
| **Depression/Anxiety** | | | | | |
| Neuroticism | 42,298 | -0.11 | 0.014 | -7.553 | 2.61×10^-13^ |
| Depression symptom | 29,125 | -0.069 | 0.009 | -7.369 | 7.98×10^-13^ |
| Anxiety symptom | 29,232 | -0.042 | 0.013 | -3.294 | 1.14×10^-03^ |
| CIDI depression | 9,960 | -0.066 | 0.015 | -4.520 | 8.86×10^-06^ |
| **Subjective well-being** | | | | | |
| Happiness: general | 29,422 | -0.048 | 0.010 | -4.728 | 4.27×10^-06^ |
| Happiness with own health | 29,501 | -0.11 | 0.010 | -11.35 | 8.11×10^-29^ |
| Belief that life is meaningful | 28,961 | 0.038 | 0.013 | 2.870 | 4.56×10^--03^ |
| **Life satisfaction** | | | | | |
| Satisfaction: health | 42,764 | -0.13 | 0.009 | -14.74 | 7.16×10^-48^ |
| Satisfaction: family relationship | 42,506 | -0.036 | 0.008 | -4.557 | 7.81×10^-06^ |
| Satisfaction: friendship | 42,384 | -0.034 | 0.008 | -4.510 | 8.86×10^-06^ |
| Satisfaction: financial situation | 42,750 | -0.052 | 0.008 | -6.697 | 8.10×10^-11^ |
| Satisfaction: job/work | 24,053 | -0.047 | 0.012 | -3.963 | 9.26×10^-05^ |
| Happiness | 42,725 | -0.064 | 0.009 | -7.361 | 7.98×10^-13^ |

| **Table S4. Brain regions showing significant correlations with grip strength** | | | | | | |
| --- | --- | --- | --- | --- | --- | --- |
| **Brain regions** | **Total No.** | ***r*** | **95% CI**  **Lower Upper** | | **T-value** | **P_FDR_** |
| R.ventral_striatum | 37,563 | 0.068 | 0.053 | 0.083 | 9.05 | 2.17×10^-17^ |
| R.hippocampus | 37,545 | 0.065 | 0.050 | 0.080 | 8.46 | 2.00×10^-15^ |
| L.ventral_striatum | 37,565 | 0.063 | 0.047 | 0.079 | 7.84 | 2.20×10^-13^ |
| L.hippocampus | 37,547 | 0.060 | 0.045 | 0.076 | 7.5 | 2.19×10^-12^ |
| R.pallidum | 37,521 | 0.055 | 0.040 | 0.069 | 7.41 | 3.58×10^-12^ |
| L.thalamus | 37,534 | 0.060 | 0.042 | 0.078 | 6.56 | 1.30×10^-09^ |
| L.parahipp_gyrus_post | 37,550 | 0.048 | 0.034 | 0.063 | 6.41 | 2.55×10^-09^ |
| L.temp_fusif_cortex_ant | 37,559 | 0.058 | 0.040 | 0.076 | 6.41 | 2.55×10^-09^ |
| L.temporal_pole | 37,549 | 0.049 | 0.034 | 0.064 | 6.36 | 3.20×10^-09^ |
| L.putamen | 37,547 | 0.052 | 0.035 | 0.069 | 5.98 | 3.15×10^-08^ |
| R.parahipp_gyrus_ant | 37,562 | 0.053 | 0.035 | 0.070 | 5.82 | 7.68×10^-08^ |
| R.temp_fusif_cortex_ant | 37,557 | 0.040 | 0.026 | 0.054 | 5.68 | 1.60×10^-07^ |
| R.putamen | 37,542 | 0.044 | 0.029 | 0.059 | 5.66 | 1.66×10^-07^ |
| L.caudate | 37,544 | 0.041 | 0.026 | 0.056 | 5.42 | 5.60×10^-07^ |
| R.amygdala | 37,548 | 0.044 | 0.028 | 0.060 | 5.42 | 5.60×10^-07^ |
| R.thalamus | 37,533 | 0.055 | 0.035 | 0.075 | 5.32 | 9.17×10^-07^ |
| R.temporal_pole | 37,556 | 0.040 | 0.025 | 0.054 | 5.23 | 1.41×10^-06^ |
| brain_stem | 37,539 | 0.044 | 0.027 | 0.062 | 4.98 | 4.96×10^-06^ |
| L.parahipp_gyrus_ant | 37,554 | 0.060 | 0.036 | 0.083 | 4.95 | 5.34×10^-06^ |
| L.cing_gyrus_ant | 37,520 | -0.038 | -0.053 | -0.023 | -4.89 | 7.16×10^-06^ |
| R.caudate | 37,546 | 0.036 | 0.021 | 0.051 | 4.76 | 1.26×10^-05^ |
| L.amygdala | 37,544 | 0.036 | 0.021 | 0.051 | 4.66 | 1.96×10^-05^ |
| R.postcent_gyrus | 37,547 | 0.038 | 0.020 | 0.056 | 4.14 | 2.12×10^-04^ |
| L.pallidum | 37,509 | 0.060 | 0.031 | 0.088 | 4.12 | 2.19×10^-04^ |
| L.occ_pole | 37,558 | 0.031 | 0.016 | 0.046 | 4.07 | 2.67×10^-04^ |
| R.front_orb_cortex | 37,553 | 0.037 | 0.018 | 0.056 | 3.82 | 7.29×10^-04^ |
| R.cing_gyrus_ant | 37,534 | -0.032 | -0.049 | -0.016 | -3.77 | 8.46×10^-04^ |
| L.front_orb_cortex | 37,551 | 0.027 | 0.013 | 0.042 | 3.76 | 8.46×10^-04^ |
| R.cerebellum_X | 37,554 | 0.042 | 0.020 | 0.064 | 3.75 | 8.54×10^-04^ |
| L.inf_temp_gyrus_ant | 37,561 | 0.028 | 0.013 | 0.043 | 3.65 | 1.21×10^-03^ |
| V_cerebellum_crus_II | 37,558 | 0.032 | 0.014 | 0.050 | 3.55 | 1.74×10^-03^ |
| R.sup_temp_gyrus_post | 37,560 | 0.028 | 0.013 | 0.044 | 3.53 | 1.82×10^-03^ |
| R.parahipp_gyrus_post | 37,553 | 0.028 | 0.012 | 0.044 | 3.47 | 2.23×10^-03^ |
| L.cerebellum_X | 37,551 | 0.041 | 0.017 | 0.064 | 3.44 | 2.41×10^-03^ |
| L.mid_temp_gyrus_ant | 37,560 | 0.035 | 0.015 | 0.056 | 3.36 | 2.97×10^-03^ |
| R.mid_temp_gyrus_post | 37,556 | 0.031 | 0.013 | 0.049 | 3.36 | 2.97×10^-03^ |
| L.cerebellum_V | 37,558 | 0.034 | 0.012 | 0.055 | 3.05 | 8.56×10^-03^ |
| L.insular_cortex | 37,540 | 0.023 | 0.008 | 0.037 | 3.03 | 8.83×10^-03^ |
| R.inf_temp_gyrus_ant | 37,559 | 0.031 | 0.011 | 0.051 | 3.02 | 8.96×10^-03^ |

| **Table S5. Brain regions showing significant correlations with grip strength after controlling for the total intracranial volume** | | | | | | | | |
| --- | --- | --- | --- | --- | --- | --- | --- | --- |
| **Brain regions** | | **Total No.** | | ***r*** | **95% CI**  **Lower Upper** | | **T-value** | **P_FDR_** |
| L.thalamus | 37,114 | | 0.061 | | 0.049 | 0.074 | 9.52 | 2.63×10^-19^ |
| R.ventral_striatum | 37,141 | | 0.068 | | 0.054 | 0.082 | 9.41 | 3.56×10^-19^ |
| R.hippocampus | 37,125 | | 0.063 | | 0.049 | 0.077 | 8.73 | 1.19×10^-16^ |
| L.ventral_striatum | 37,143 | | 0.061 | | 0.046 | 0.075 | 8.18 | 1.03×10^-14^ |
| R.thalamus | 37,113 | | 0.055 | | 0.042 | 0.068 | 8.13 | 1.19×10^-14^ |
| L.hippocampus | 37,127 | | 0.061 | | 0.046 | 0.076 | 8.02 | 2.48×10^-14^ |
| L.temporal_pole | 37,128 | | 0.047 | | 0.034 | 0.060 | 7.16 | 1.61×10^-11^ |
| brain_stem | 37,119 | | 0.044 | | 0.032 | 0.056 | 7.13 | 1.76×10^-11^ |
| R.parahipp_gyrus_ant | 37,140 | | 0.050 | | 0.036 | 0.064 | 6.98 | 4.49×10^-11^ |
| L.temp_fusif_cortex_ant | 37,138 | | 0.054 | | 0.039 | 0.070 | 6.72 | 2.58×10^-10^ |
| R.pallidum | 37,100 | | 0.053 | | 0.037 | 0.069 | 6.61 | 4.91×10^-10^ |
| R.putamen | 37,121 | | 0.043 | | 0.030 | 0.056 | 6.5 | 9.21×10^-10^ |
| L.parahipp_gyrus_post | 37,129 | | 0.048 | | 0.033 | 0.063 | 6.41 | 1.59×10^-09^ |
| R.temp_fusif_cortex_ant | 37,136 | | 0.040 | | 0.027 | 0.054 | 5.92 | 3.30×10^-08^ |
| L.parahipp_gyrus_ant | 37,133 | | 0.056 | | 0.037 | 0.074 | 5.84 | 4.94×10^-08^ |
| L.caudate | 37,123 | | 0.039 | | 0.026 | 0.053 | 5.68 | 1.15×10^-07^ |
| L.putamen | 37,126 | | 0.047 | | 0.031 | 0.063 | 5.64 | 1.38×10^-07^ |
| R.amygdala | 37,127 | | 0.042 | | 0.026 | 0.058 | 5.12 | 2.41×10^-06^ |
| L.mid_temp_gyrus_ant | 37,138 | | 0.035 | | 0.021 | 0.049 | 5.04 | 3.38×10^-06^ |
| L.occ_pole | 371,36 | | 0.033 | | 0.02 | 0.047 | 4.8 | 1.09×10^-05^ |
| R.temporal_pole | 371,35 | | 0.037 | | 0.022 | 0.052 | 4.74 | 1.41×10^-05^ |
| R.caudate | 371,25 | | 0.033 | | 0.019 | 0.047 | 4.65 | 2.09×10^-05^ |
| R.front_orb_cortex | 371,32 | | 0.035 | | 0.020 | 0.049 | 4.61 | 2.49×10^-05^ |
| L.front_orb_cortex | 371,31 | | 0.027 | | 0.016 | 0.039 | 4.57 | 2.80×10^-05^ |
| R.mid_temp_gyrus_post | 37,136 | | 0.030 | | 0.017 | 0.043 | 4.49 | 3.98×10^-05^ |
| R.postcent_gyrus | 37,127 | | 0.035 | | 0.018 | 0.053 | 4.05 | 2.73×10^-04^ |
| L.cing_gyrus_ant | 37,100 | | -0.037 | | -0.055 | -0.019 | -4.02 | 3.01×10^-04^ |
| R.cerebellum_X | 37,133 | | 0.038 | | 0.019 | 0.058 | 3.96 | 3.78×10^-04^ |
| L.lingual_gyrus | 37,134 | | 0.027 | | 0.013 | 0.040 | 3.89 | 4.74×10^-04^ |
| L.inf_temp_gyrus_ant | 37,139 | | 0.028 | | 0.014 | 0.043 | 3.85 | 5.49×10^-04^ |
| R.supramarg_gyrus_post | 37,135 | | 0.034 | | 0.016 | 0.052 | 3.79 | 6.61×10^-04^ |
| R.parahipp_gyrus_post | 37,131 | | 0.028 | | 0.014 | 0.043 | 3.79 | 6.61×10^-04^ |
| L.cerebellum_X | 37,131 | | 0.037 | | 0.018 | 0.056 | 3.78 | 6.61×10^-04^ |
| L.mid_temp_gyrus_post | 37,135 | | 0.025 | | 0.012 | 0.039 | 3.65 | 1.08×10^-03^ |
| V_cerebellum_crus_II | 37,137 | | 0.033 | | 0.015 | 0.050 | 3.63 | 1.14×10^-03^ |
| R.supramarg_gyrus_ant | 37,133 | | 0.026 | | 0.012 | 0.041 | 3.56 | 1.42×10^-03^ |
| L.insular_cortex | 37,120 | | 0.021 | | 0.009 | 0.033 | 3.49 | 1.85×10^-03^ |
| R.sup_temp_gyrus_post | 37,138 | | 0.025 | | 0.010 | 0.040 | 3.34 | 3.10×10^-03^ |
| R.occ_pole | 37,133 | | 0.026 | | 0.011 | 0.041 | 3.32 | 3.15×10^-03^ |
| R.planum_temporale | 37,137 | | 0.023 | | 0.009 | 0.037 | 3.27 | 3.75×10^-03^ |
| R.cing_gyrus_ant | 37,115 | | -0.033 | | -0.054 | -0.013 | -3.24 | 4.07×10^-03^ |
| R.cerebellum_crus_II | 37,124 | | 0.047 | | 0.018 | 0.076 | 3.22 | 4.22×10^-03^ |
| L.precun_cortex | 37,132 | | 0.020 | | 0.008 | 0.032 | 3.19 | 4.63×10^-03^ |
| R.lingual_gyrus | 37,131 | | 0.023 | | 0.008 | 0.037 | 3.05 | 6.95×10^-03^ |
| R.parietal_operc_cortex | 37,136 | | 0.022 | | 0.008 | 0.035 | 3.05 | 6.95×10^-03^ |
| L.amygdala | 37,122 | | 0.032 | | 0.012 | 0.053 | 3.05 | 6.95×10^-03^ |
| L.cerebellum_V | 37,136 | | 0.030 | | 0.01 | 0.049 | 3.0 | 8.02×10^-03^ |
| L.paracing_gyrus | 37,129 | | 0.020 | | 0.007 | 0.033 | 2.98 | 8.48×10^-03^ |
| L.front_med_cortex | 37,131 | | 0.022 | | 0.008 | 0.037 | 2.96 | 8.82×10^-03^ |
| V_cerebellum_VI | 37,136 | | 0.029 | | 0.009 | 0.048 | 2.93 | 9.41×10^-03^ |
| R.heschl_gyrus | 37,135 | | 0.019 | | 0.006 | 0.032 | 2.91 | 1.00×10^-02^ |

| **Table S6. Results of the mediation analyses between grip strength, phenotypes, and mean grey matter volume in all subjects** | | | | | |
| --- | --- | --- | --- | --- | --- |
|  | Total No. | Prop.  Mediated (%) | 95% CI  Lower-Upper | | P_FDR_ |
| Prospective memory | 35,149 | 2.42 | 1.30 | 3.90 | <2×10^-4^ |
| Reaction time | 34,933 | 1.64 | 1.03 | 2.37 | <2×10^-4^ |
| Numeric memory | 25,367 | 21.83 | 13.80 | 41.88 | <2×10^-4^ |
| Trail making: part A | 24,741 | 13.23 | 8.73 | 21.76 | <2×10^-4^ |
| Symbol-digit substitution: attempted | 24,771 | 15.58 | 9.66 | 29.87 | <2×10^-4^ |
| Symbol-digit substitution: corrected | 24,771 | 15.26 | 9.31 | 29.51 | <2×10^-4^ |
| Matrix pattern completion | 24,765 | 12.31 | 8.65 | 17.90 | <2×10^-4^ |
| Tower rearranging | 24,557 | 9.83 | 5.97 | 17.95 | <2×10^-4^ |
| Verbal declarative memory | 25,024 | 5.21 | 2.46 | 10.55 | <2×10^-4^ |
| Pairs-matching 1: error made | 35,166 | 7.28 | 3.90 | 15.75 | <2×10^-4^ |
| Pairs-matching 2: error made | 35,166 | 4.87 | 1.84 | 11.80 | <2×10^-4^ |
| Pairs-matching 3: error made | 24,492 | -1.71 | -13.23 | 3.49 | 0.439 |
| Pairs-matching 1: time | 34,563 | 6.43 | 4.40 | 9.37 | <2×10^-4^ |
| Pairs-matching 2: time | 34,571 | 9.01 | 5.36 | 17.99 | <2×10^-4^ |
| Neuroticism | 36,688 | 0.66 | -0.13 | 1.55 | 0.135 |
| Depression symptom | 25,387 | 0.94 | -0.31 | 2.35 | 0.166 |
| Anxiety symptom | 25,486 | 0.53 | -1.47 | 2.71 | 0.594 |
| CIDI depression | 8,749 | 1.62 | -0.33 | 4.99 | 0.135 |
| Happiness: general | 25,637 | -2.80 | -5.81 | -0.84 | 0.005 |
| Happiness with own health | 25,709 | 1.40 | 0.55 | 2.42 | 1.35×10^-3^ |
| Belief that life is meaningful | 25,244 | -0.25 | -3.94 | 3.15 | 0.871 |
| Satisfaction: health | 37,078 | 1.74 | 1.02 | 2.60 | <2×10^-4^ |
| Satisfaction: family relationship | 36,872 | -1.56 | -4.54 | 0.54 | 0.200 |
| Satisfaction: friendship | 36,758 | -4.93 | -9.90 | -2.34 | <2×10^-4^ |
| Satisfaction: financial situation | 37,077 | 6.10 | 3.77 | 9.67 | <2×10^-4^ |
| Satisfaction: job/work | 21,095 | -1.53 | -5.90 | 1.25 | 0.292 |
| Happiness | 37,050 | -2.15 | -4.06 | -0.67 | 0.003 |


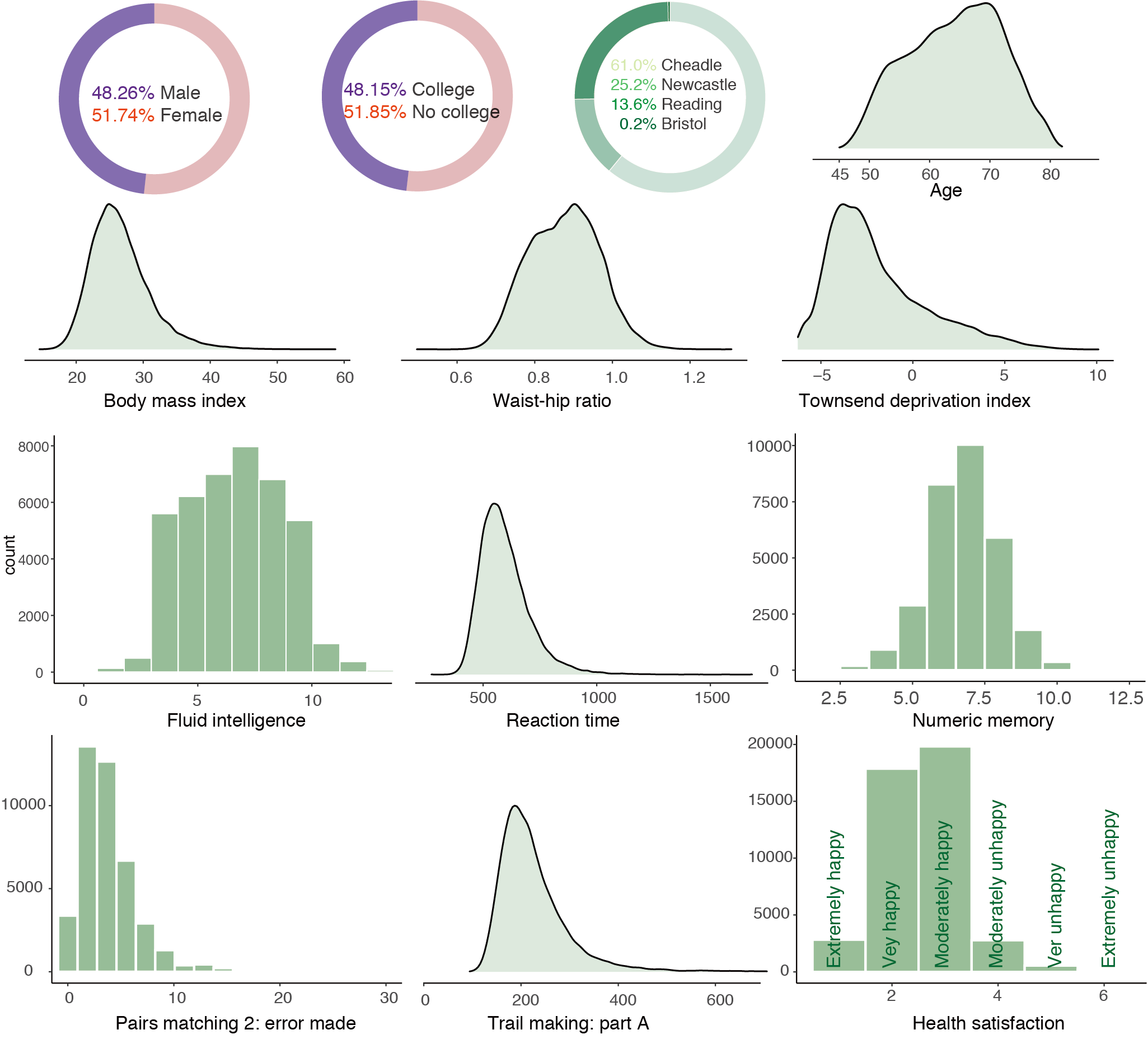


**Figure S1.** A brief summary of the population characteristics of all participants used in the current study.


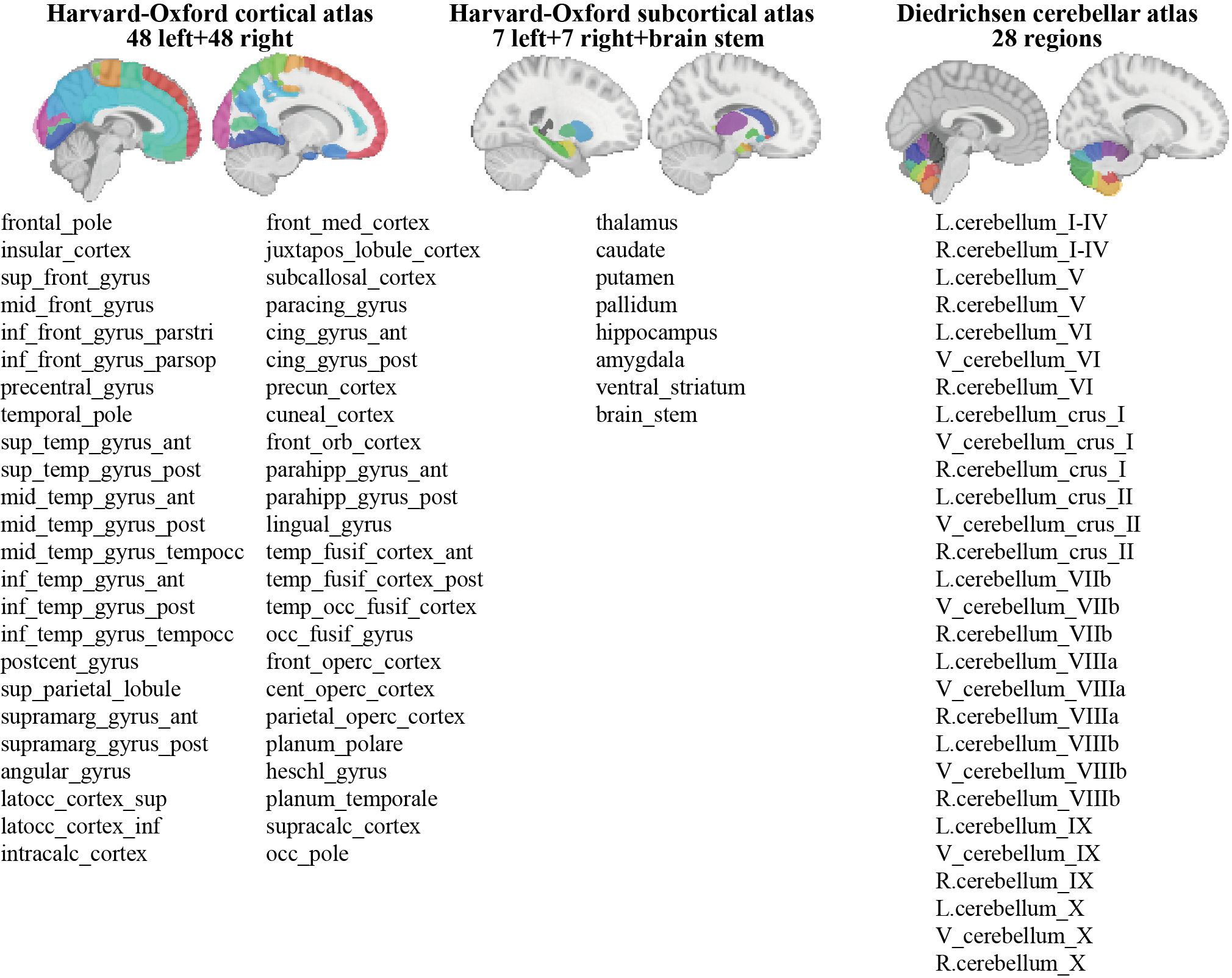


**Figure S2.** The 139 brain regions and their names. Specifically, tissue-type segmentation was applied using FAST (FMRIB’s Automated Segmentation Tool), and subcortical structures were modeled using FIRST (FMRIB’s Integrated Registration and Segmentation Tool). An extensive overview of the data preprocessing carried out can be found at <https://biobank.ctsu.ox.ac.uk/crystal/crystal/docs/brain_mri.pdf>. The 139 brain regions include 96 cortical and 15 subcortical regions based on Harvard-Oxford atlas (https://fsl.fmrib.ox.ac.uk/fsl/fslwiki/Atlases), and 28 cerebellar regions based on Diedrichsen cerebellar atlas (http://www.diedrichsenlab.org/imaging/propatlas.htm).


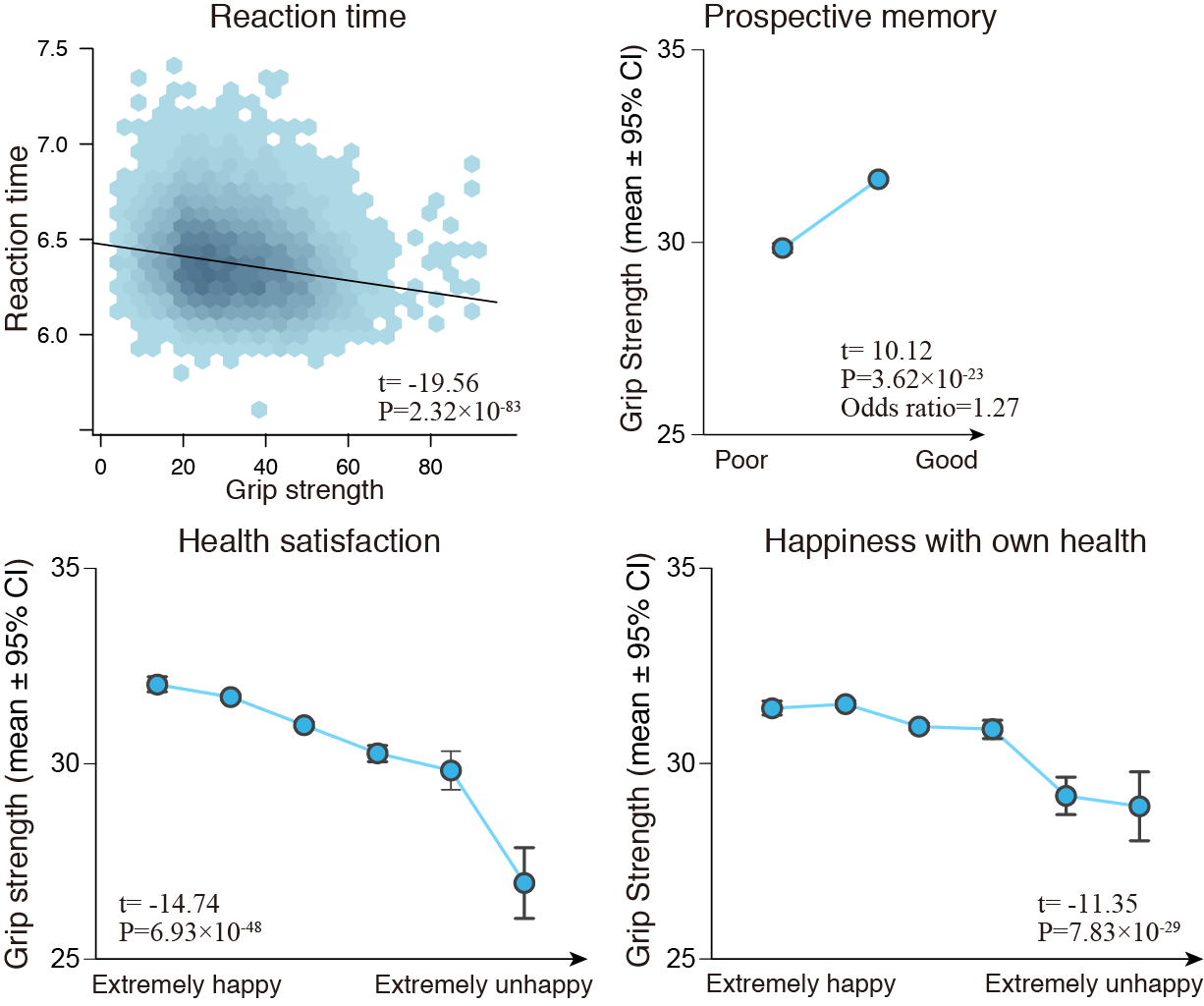


**Figure S3.** The top four behavioral outcomes showing the strongest associations with grip strength. Of all 30 behavioral outcomes, 27 were significantly correlated with grip strength while controlling for confounding variables (FDR corrected P<0.05). The strongest behavior effect was for reaction time, followed by health satisfaction, happiness with one's own health, and prospective memory.


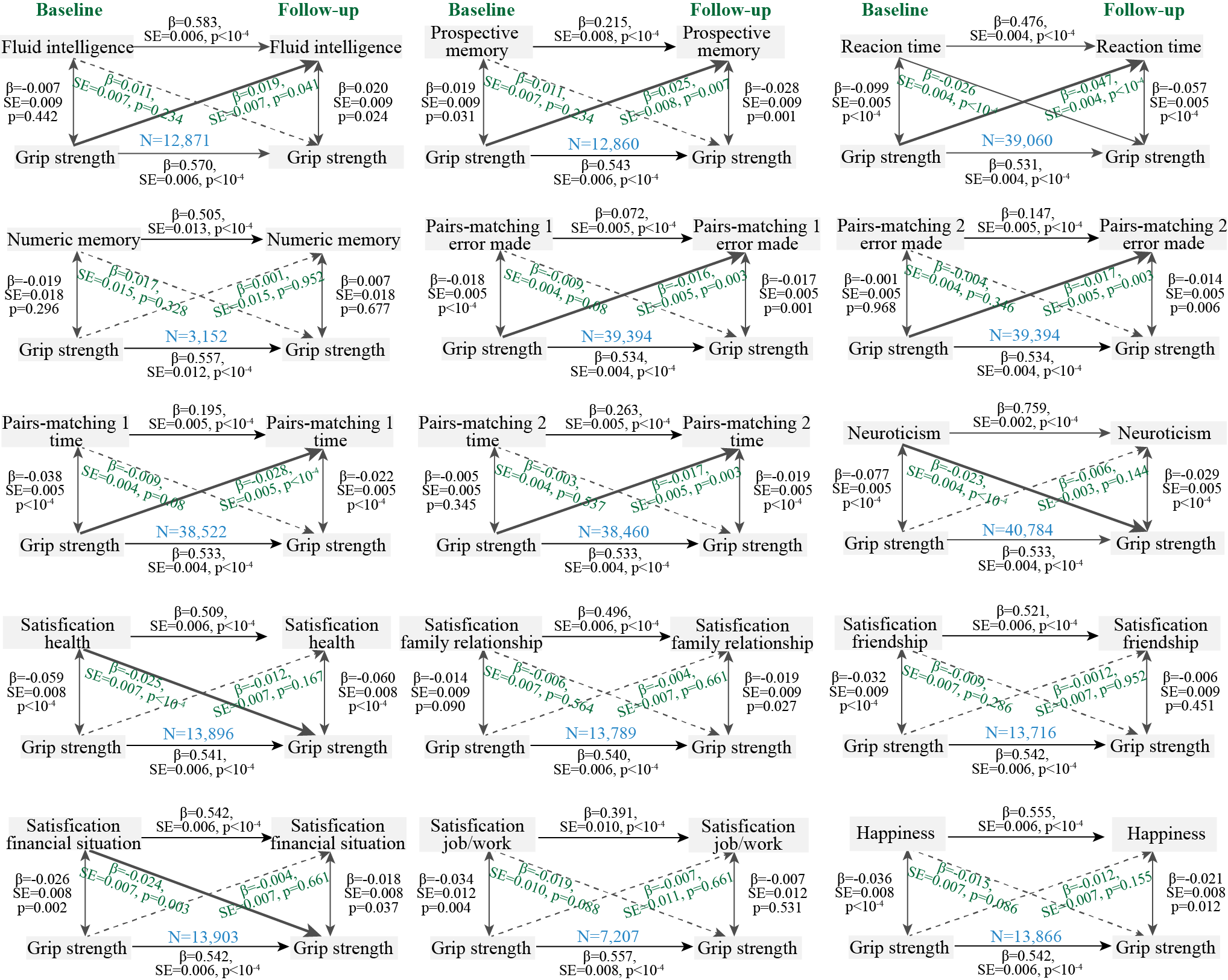


**Figure S4.** Results of the classic two-wave cross-lagged panel model for 15 behavioral phenotypes that have complete data at two time points.


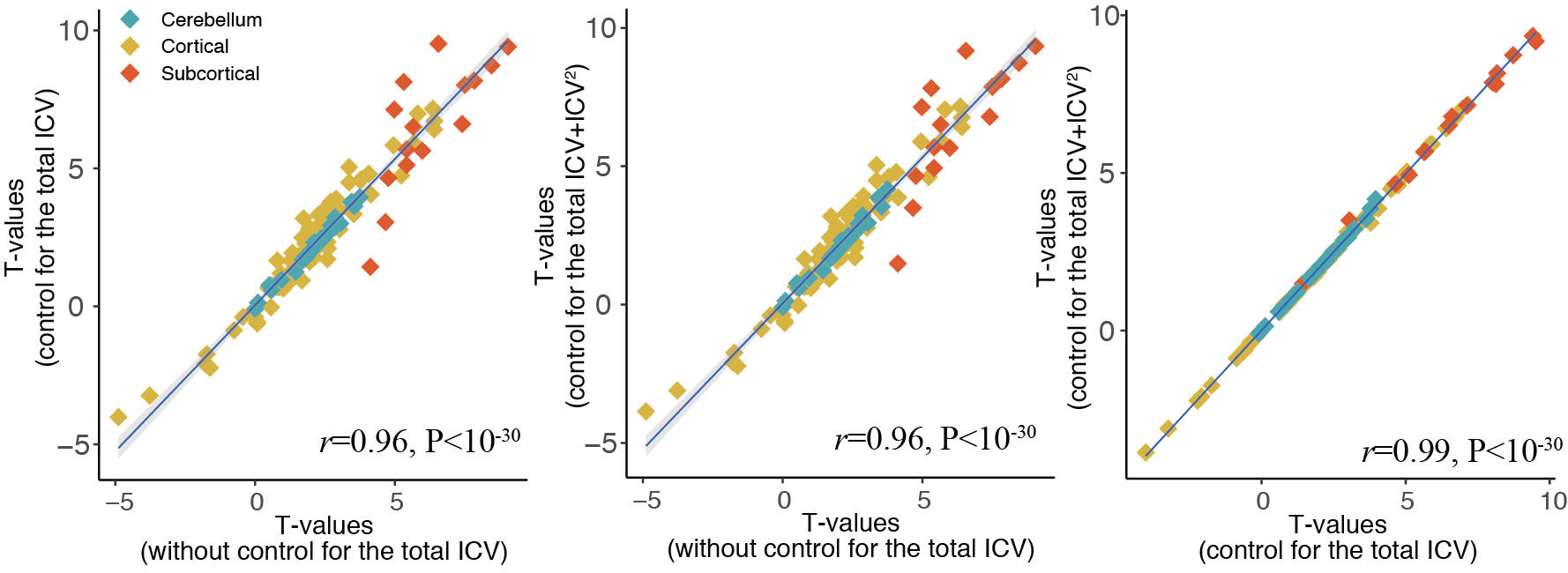


**Figure S5.** The correlation of association maps (T-maps) between the cases with and without including the total intracranial volume (ICV) and the squared ICV as covariates in examining the association of grip strength with regional grey matter volume across 139 regions.


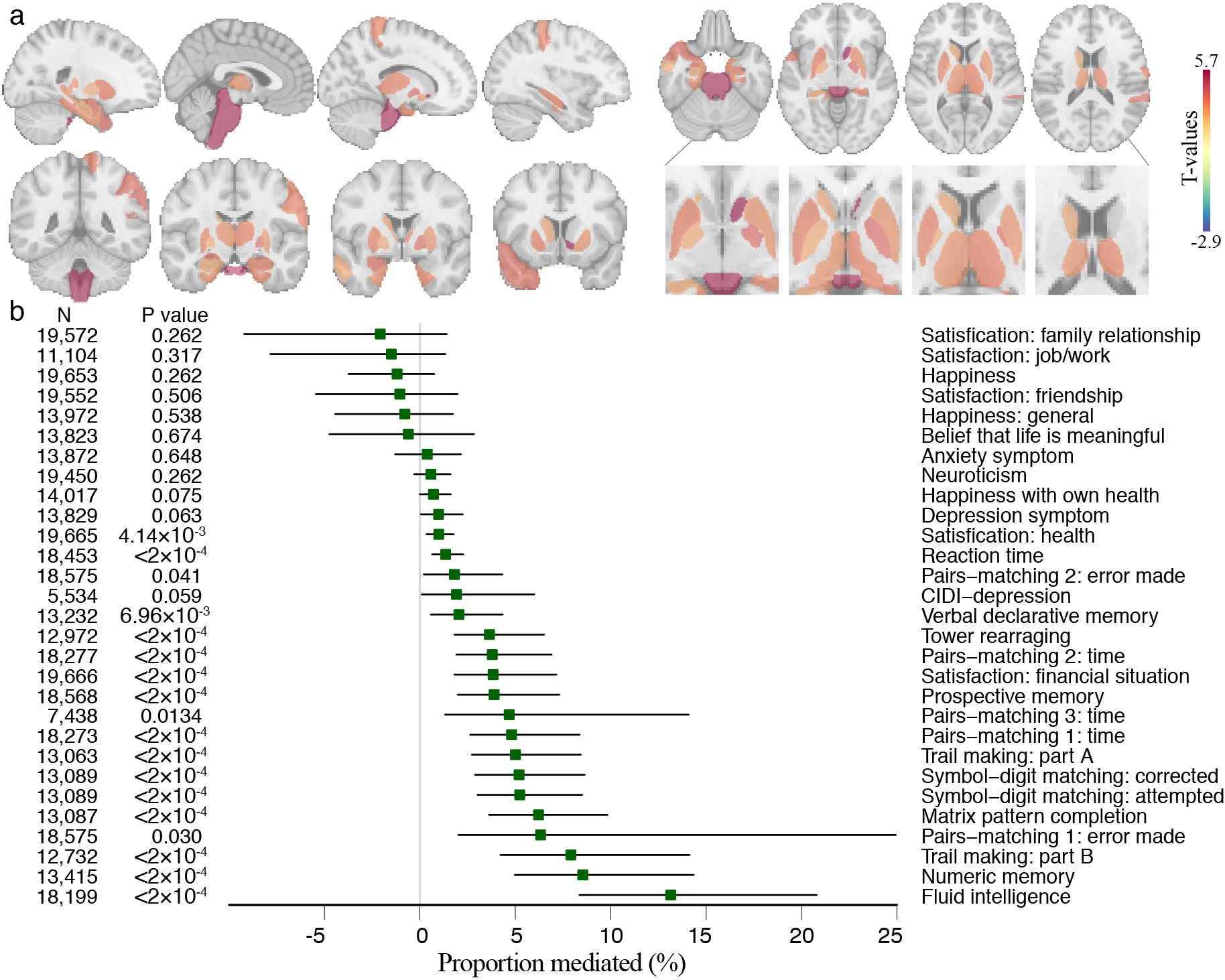


**Figure S6.** Regional distribution of associations between grey matter volume and grip strength and the mediation effect of mean GMV in females. **(a)** Brain regions showing the highest correlations with grip strength primarily included: the right ventral striatum, brain stem, right pallidum, right posterior supramarginal gyrus, left temporal pole, left amygdala, left putamen, and right hippocampus. (**b**) Mediation effect of mean GMV on the association between grip strength and behavioral outcomes in females. The proportion of variance explained by the mediation as well as the lower and upper bound of 95% confidence interval was shown. Of all 29 behavioral outcomes significantly associated with grip strength, mediation analyses revealed a significant indirect effect (a×b) of the mean GMV in 18 outcomes (FDR corrected P<0.05).


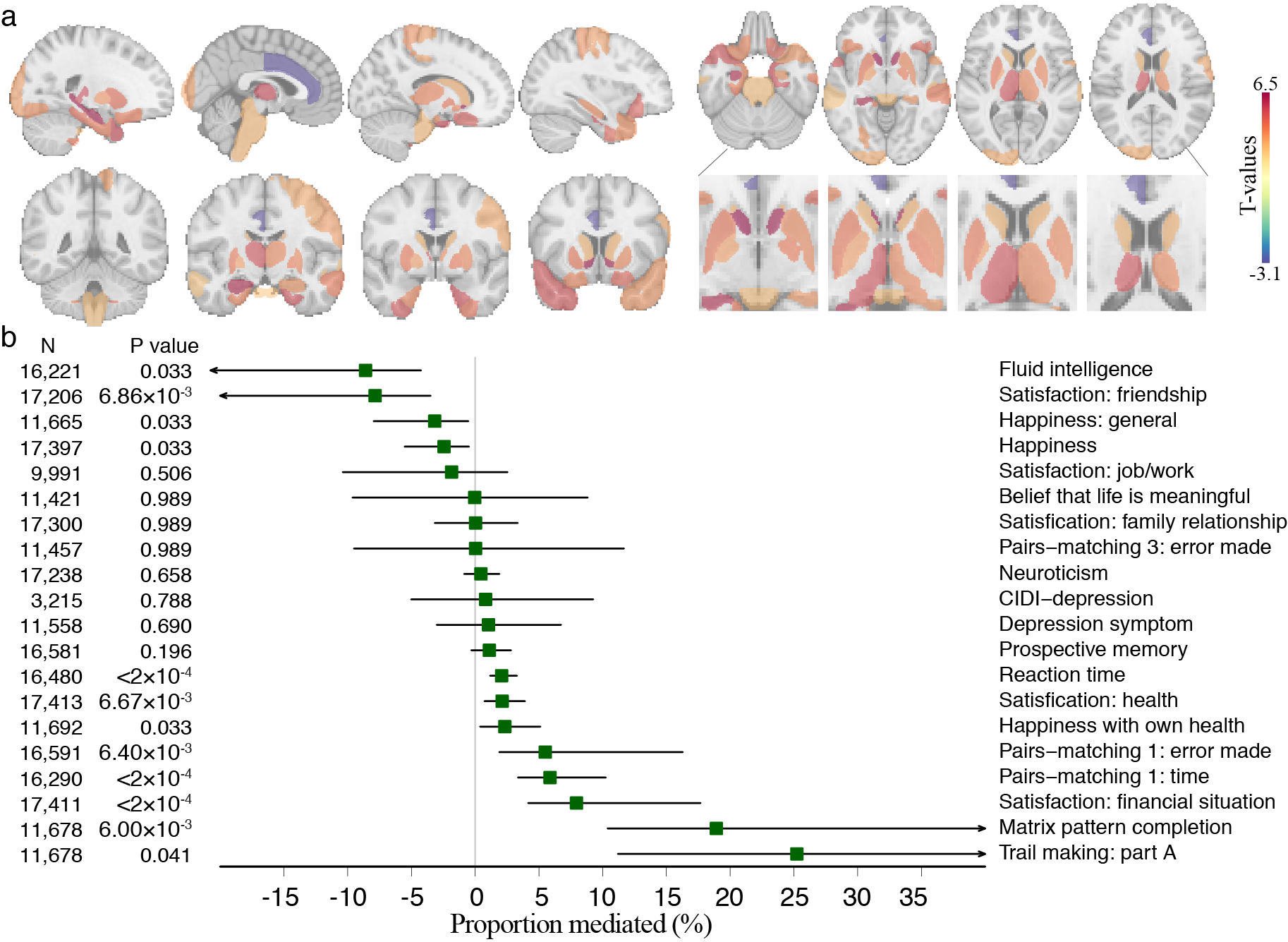


**Figure S7.** Regional distribution of associations between grey matter volume and grip strength and the mediation effect of mean GMV in males. **(a)** Brain regions showing the highest correlations with grip strength primarily included: the ventral striatum, left hippocampus, left thalamus, parahippocampal gyrus, left temporal pole, anterior temporal fusiform cortex, right orbitofrontal cortex, and left putamen. (**b**) Mediation effect of mean GMV on the association between grip strength and behavioral outcomes in males. The proportion of variance explained by the mediation as well as the lower and upper bound of 95% confidence interval was shown. Of all 20 behavioral outcomes significantly associated with grip strength, mediation analyses revealed a significant indirect effect (a×b) of the mean GMV in 8 outcomes (FDR corrected P<0.05).


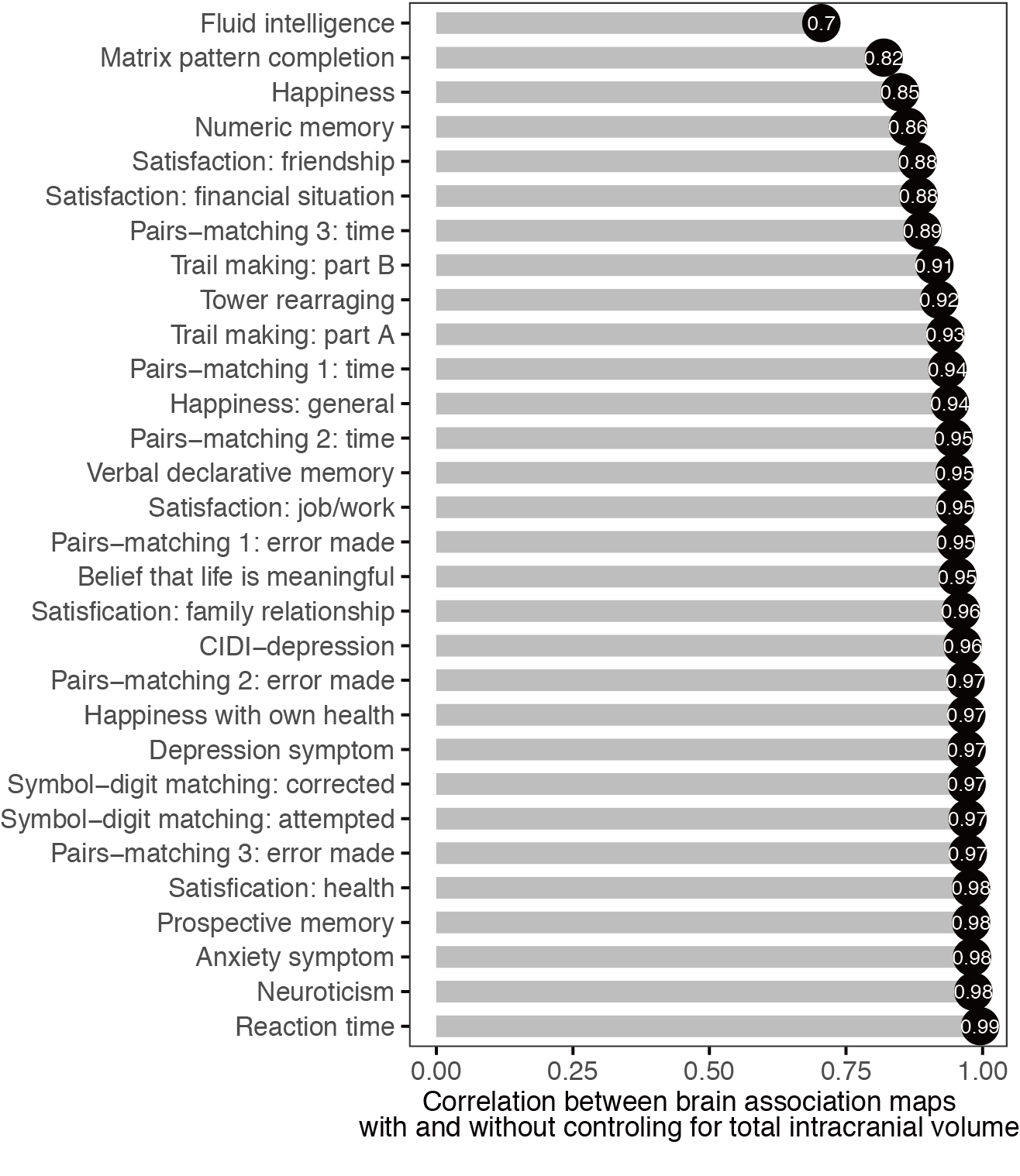


**Figure S8**. The correlation of association-maps between the cases with and without including the total intracranial volume as a covariate in examining the association of grey matter volumes with behavioral outcomes across 139 regions.


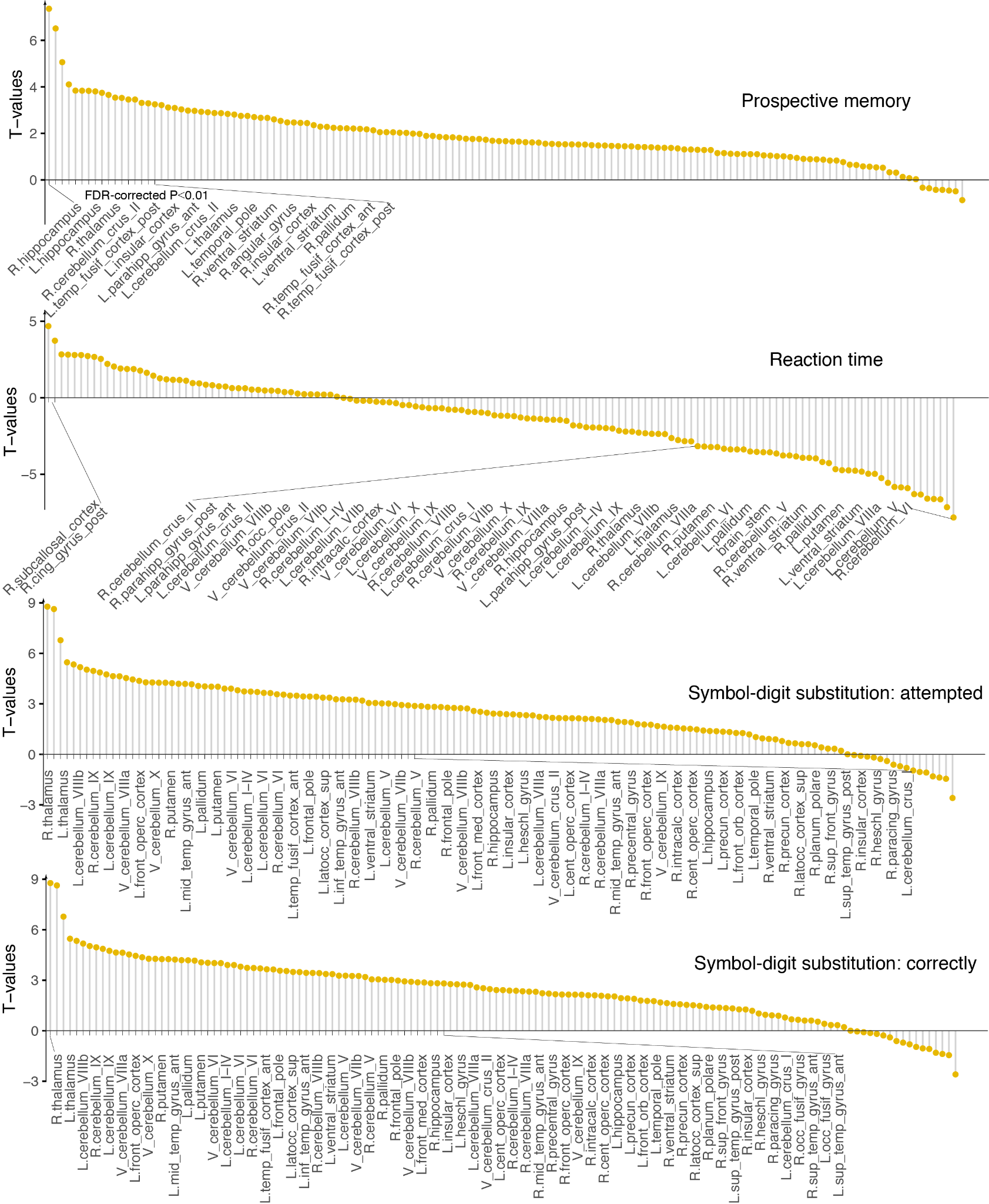


**Figure S9.** **Regional distribution of associations between grey matter volume and four representative behavioral phenotypes.** The top 4 behavioral phenotypes showing the highest similarities of association map with grip strength were prospective memory, reaction time, symbol-digit substitution: attempted, and symbol-digit substitution: correctly.


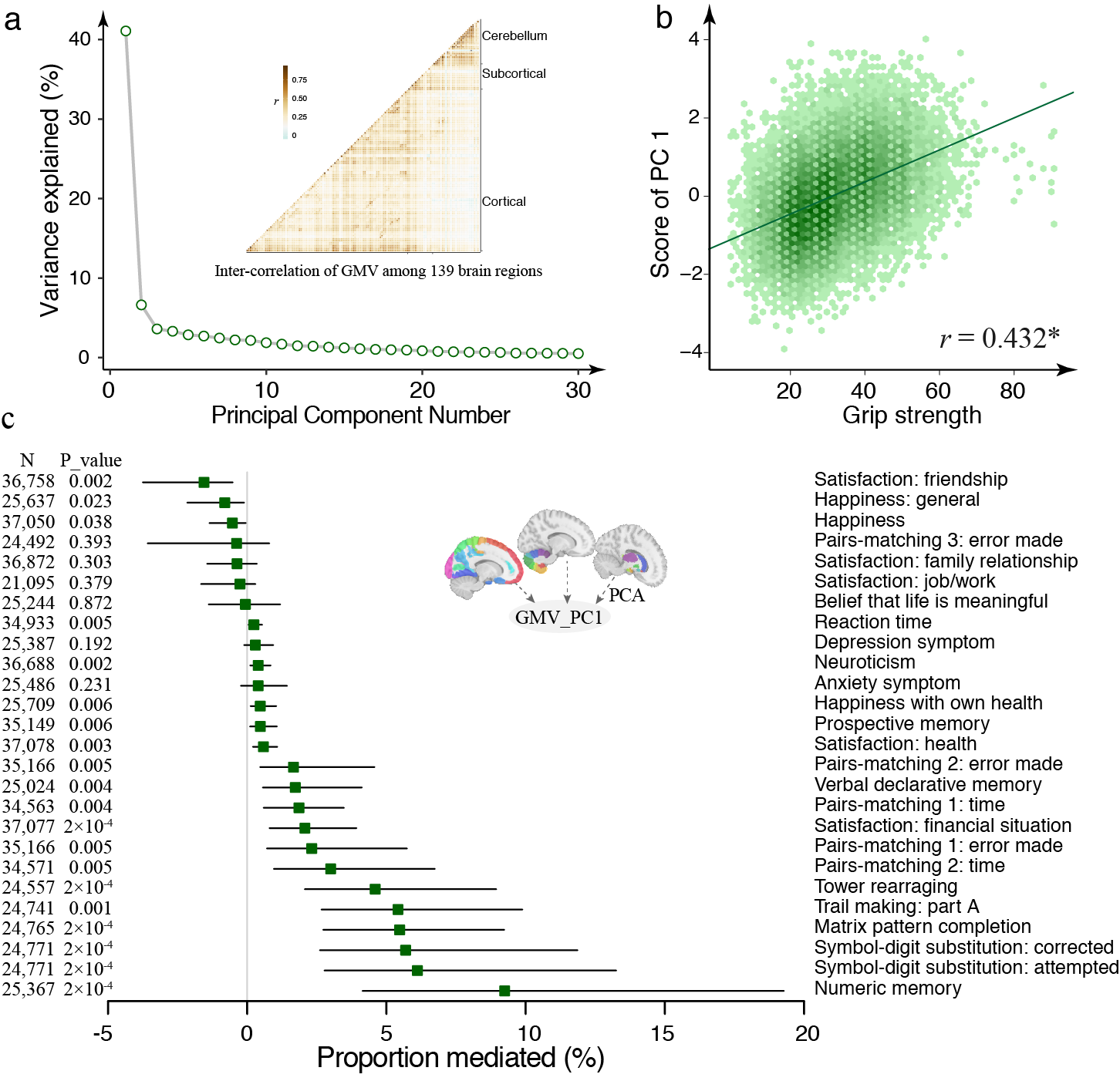


**Figure S10. Mediation effects of the first principal component of 139 regional GMV on the association between grip strength and behavioral outcomes.** Considering the high correlations between regional GMVs, we performed a principal component analysis (PCA) and extracted the first principal component as a general representation of GMV across the brain (GMV_PC1). (**a**) Scree plot showing the variance explained by each component in the principal component analysis based on 139 regional GMV. (**b**) The first latent component (GMV_PC1) was highly correlated with grip strength (*r*=0.432, P<10^-30^). (**c**) Mediation effect of GMV_PC1 on the association between grip strength and behavioral outcomes. The proportion of variance explained by the mediation as well as the lower and upper bound of 95% confidence interval was shown. a×b: the indirect effect; c: the total effect; c’: the direct effect.
